# A Review with Updated Perspectives on the Antiviral Potentials of Traditional Medicinal Plants and Their Prospects in Antiviral Therapy

**DOI:** 10.3390/life12081287

**Published:** 2022-08-22

**Authors:** Nur Fadlin Saifulazmi, Emelda Rosseleena Rohani, Sarahani Harun, Hamidun Bunawan, Hamizah Shahirah Hamezah, Nor Azlan Nor Muhammad, Kamalrul Azlan Azizan, Qamar Uddin Ahmed, Sharida Fakurazi, Ahmed Mediani, Murni Nazira Sarian

**Affiliations:** 1 Faculty of Science, Universiti Malaya, Lembah Pantai, Wilayah Persekutuan, Kuala Lumpur 56300, Selangor, Malaysia; 2 Institute of Systems Biology (INBIOSIS), Universiti Kebangsaan Malaysia, Bangi 43600, Selangor, Malaysia; 3 Drug Discovery and Synthetic Chemistry Research Group, Department of Pharmaceutical Chemistry, Kulliyyah of Pharmacy, International Islamic University Malaysia, Kuantan 25200, Pahang, Malaysia; 4 Laboratory of Natural Medicines and Product Research, Institute of Bioscience, Universiti Putra Malaysia, Serdang 43400, Selangor, Malaysia; 5 Department of Human Anatomy, Faculty of Medicine and Health Sciences, Universiti Putra Malaysia, Serdang 43400, Selangor, Malaysia

**Keywords:** medicinal plants, virus infections, antiviral activities, bioactive compounds

## Abstract

Exploration of the traditional medicinal plants is essential for drug discovery and development for various pharmacological targets. Various phytochemicals derived from medicinal plants were extensively studied for antiviral activity. This review aims to highlight the role of medicinal plants against viral infections that remains to be the leading cause of human death globally. Antiviral properties of phytoconstituents isolated from 45 plants were discussed for five different types of viral infections. The ability of the plants’ active compounds with antiviral effects was highlighted as well as their mechanism of action, pharmacological studies, and toxicological data on a variety of cell lines. The experimental values, such as IC_50_, EC_50_, CC_50_, ED_50_, TD_50_, MIC_100_, and SI of the active compounds, were compiled and discussed to determine their potential. Among the plants mentioned, 11 plants showed the most promising medicinal plants against viral infections. *Sambucus nigra* and *Clinacanthus nutans* manifested antiviral activity against three different types of viral infections. *Echinacea purpurea, Echinacea augustofolia, Echinacea pallida, Plantago major, Glycyrrhiza uralensis, Phyllanthus emblica, Camellia sinensis,* and *Cistus incanus* exhibited antiviral activity against two different types of viral infections. Interestingly, *Nicotiana benthamiana* showed antiviral effects against mosquito-borne infections. The importance of phenolic acids, alkamides, alkylamides, glycyrrhizin, epicatechin gallate (ECG), epigallocatechin gallate (EGCG), epigallocatechin (EGC), protein-based plant-produced ZIKV Envelope (PzE), and anti-CHIKV monoclonal antibody was also reviewed. An exploratory approach to the published literature was conducted using a variety of books and online databases, including Scopus, Google Scholar, ScienceDirect, Web of Science, and PubMed Central, with the goal of obtaining, compiling, and reconstructing information on a variety of fundamental aspects, especially regarding medicinal plants. This evaluation gathered important information from all available library databases and Internet searches from 1992 to 2022.

## 1. Introduction

**Figure 1:**
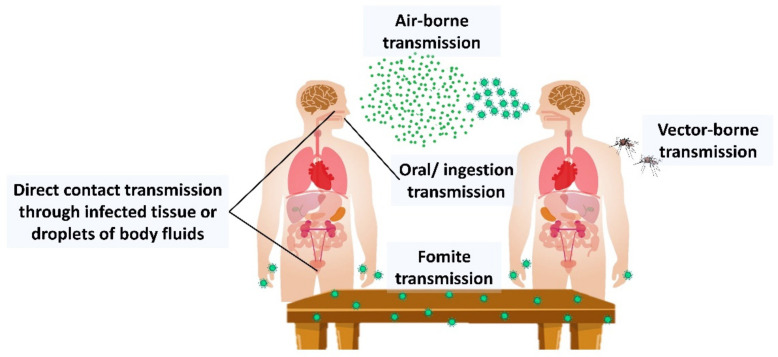
Virus infection route of transmission.

Viral infections continue to cause health threats worldwide even with the development of various types of vaccines. The emergence of new diseases caused by viral infections, such as COVID-19, has eliminated people’s assurance that infectious diseases may be effortlessly eradicated. Viral infections are the most common cause of infectious disease transmitted within close personal contact in an indoor environment. These infections are airborne via droplet or direct and indirect contact transmissions (Figure 1). The demand for antiviral properties from many sources was necessitated by the emergence of new viral agents and viral resistance mutation [1].

Traditional medicines are still widely used in most countries around the world, and their use is on the rise. Biologically active compounds isolated from medicinal plants are envisaged as new safe drugs and can be a key to finding low-cost treatments with fewer complications in curing many diseases. Traditional ethnomedicinal applications of a wide diversity of plants are now being used as a powerful tool for disease prevention [2]. Approximately 30% of the pharmaceutical market and 11% of essential drugs (drugs intended for primary care) are plant-based [3]. Depleted efficacy and emergence cases of contraindications due to the usage of synthetic drugs had increased the utilization of natural drugs [4]. The use of native plants daily for food and as herbal remedies by tribal, rural people in certain places and ancient people have elevated today’s advanced procedure of the plants’ processing and usage [5]. However, there is no ideal extraction method for all plant species, extractions using aqueous and organic solvents were commonly favored [6,7].

The antiviral properties of medicinal plants require extra exploration to be utilized as prevention in viral disease treatments [8]. Medicinal plants have a wide range of protective abilities to combat virus infections at various stages of the viral life cycle [9]. Phytochemical and pharmacological studies were conducted on traditional plants for medicinal applications to validate their therapeutic efficacies. Hence, this review aims to search for antiviral potential from plants around the globe to cope with virus infections, especially common colds, influenza, sexually transmitted diseases, dermatology related viruses, and mosquito-borne viruses. Hundreds of plants were identified, however only 45 plants from a few families have been thoroughly discussed due to their potent antiviral effects.

A virus is composed of DNA or RNA and surrounded by a protein coat. Viral infections occur when viruses invade and alter the genetic materials of healthy living cells, forcing them to manufacture more a-like viruses. People obtain viruses by swallowing, inhalation of them, through insects’ bites, or through sexual interaction. Commonly, viral infections are associated with respiratory organs (nose, throat, and lungs), leading to common cold or influenza (flu) [10]. Viruses are also found in infected blood, body fluids, cells, and tissue of several body systems, such as reproductive systems, integumentary, and gastrointestinal systems [11]. Throughout these few decades, antiviral drugs were developed to impede viruses’ activity, thus preventing further infection by engaging the PBMC proliferative cycle and proliferation of cell RNA and proteins [12,13,14]. Eventually, patients with severe disease development of viral infections due to the extensive generation of genetic variations by virus replication emerged to be more resistant to antiviral drugs [15]. An analysis of the known antiviral medications’ mechanisms of action revealed that they could decrease the virus’ adsorption or diffusion into the cell as well as the virus’ deproteinization process in the cell. They can also boost the cell’s resistance to a virus. Antiviral medications that directly target viruses include attachment inhibitors, entrance inhibitors, uncoating inhibitors, polymerase inhibitors, protease inhibitors, inhibitors of nucleoside and nucleotide reverse transcriptase, and integrase inhibitors. Researchers are encouraged to evaluate the potential of antiviral treatments by traditional therapy of medicinal plants as they showed promising antiviral mechanisms of action [6]. Table 1 summarizes the mechanism of action of common antiviral drugs. 

## 2. Medicinal Plants for Treating Common Cold

Rhinovirus (RV), respiratory syncytial virus (RSV), enterovirus, adenovirus, and human parainfluenza virus (HPIV) are some of the most important viruses that are generally considered to be the cause of common colds in humans [24,25,26]. More than 50% of all common colds are caused by rhinoviruses and coronaviruses, which cause significant morbidity in immunocompromised people and in patients with underlying chronic medical or immunosuppressed conditions [26]. Four human coronaviruses (HCoV) are known to cause common cold-like symptoms, which are HCoV-229E, HCoV-OC43, HCoV-NL63, and HCoV-HKU1 [27]. Parainfluenza viruses are single-stranded, enveloped RNA viruses of the paramyoviridaie family that cause 5% of the common cold, which is the same as rhinovirus. There are four serotypes of HPIV, which are HPIV1, HPIV2, HPIV3, and HPIV4. In addition, 47 serotypes of adenoviruses are associated with human infections and are known to be the cause of around 10% of all common colds in children [24,28]. Enteroviruses are small non-enveloped viruses and are members of the picornavirus family. Other than causing common colds, enteroviruses also cause encephalitis, myocarditis, poliomyelitis, acute heart failure, and sepsis [29]. Some symptoms of the common cold would typically include a running nose, congestion, sneezing, weakened sense of taste and smell, scratchy throat, and cough [30]. Tropical, medicinal plants that have been traditionally used in treating the common cold are described below. Table 2 summarizes the medicinal plants for treating the common cold.

### 2.1. Echinacea spp.

Echinacea or also known as coneflowers is the name of a flowering group of plants native that are to North America. Three out of nine commonly recognized types of Echinacea (*E. angustifolia, E. pallida, and E. purpurea*) were reported to contain herbal remedy ingredients to treat common colds [35]. The treatment of Echinaforce (a standardized preparation extracted from freshly harvested *E. purpurea* showed a small reduction in virus titered with the highest dose of 50 μg/mL, including its root with a *v*/*v* 65% ethanol solution) to post-infection cell lines with HCoV-229E [32]. HCoV-229E was irreversibly inactivated when exposed to Echinaforce with 50% inhibitory concentration (IC_50_) 3.2 μg/mL, which was shown through MTT assay on Huh-7, Vero, and Vero E6 cells [32]. A randomized, double-blind, placebo-controlled clinical trial was used to evaluate the ability of *E. purpurea* to prevent infection caused by rhinovirus type 39 (RV-39) [36]. According to a study, among 48 patients, 59% of the patients who were infected and given pressed juice of the aerial parts of *E. purpurea* placed in a 22% alcohol base developed cold, compared to 86% of the placebo recipients (*p* = 0.0883, by Fisher’s exact test) [37]. On the other hand, *Echinacea pallida* var. *angustifolia* displayed significant anti-rhinovirus (RV) activity. Extraction of *E. pallida* var. angustifolia root in ethanol 70%, hexane, and ethyl acetate showed a positive anti-RV activity with MIC_100_ (minimum concentration required to inactivate 100% virus) of 62, 69, and 85 μg/mL, respectively. This study concluded that alkamide constituents of *E. pallida* var. *angustifolia* were activated to some degree against rhinovirus as alkamide-rich hexane fraction showed a lower MIC_100_ than ethyl acetate fraction, which contained a relatively lesser alkamide [37].

### 2.2. Sambucus spp.

*Sambucus nigra* (common names include elder, elderberry, and black elder) is a species of flowering plant found in the family of Adoxaceae that are mostly found in Europe and North America. Recently, this plant was shown to reduce the incidence of cold as well as shorten the duration of cold and flu symptoms [38]. There have been four clinical trials of elderberries conducted so far, and they managed to reduce the cold duration and severity (fever, pain, congestion, cough). Elder flowers were approved by the German Federal Institute for Drugs and Medical Devices to be used against colds [39]. However, *S. nigra* extract-treated cells showed no detectable viral cytopathic effect (CPE) at a multiplicity of infection (MOI) bronchitis virus (IBV, a pathogenic chicken coronavirus) of 0.1 and a reduction in virus titers by six orders of magnitude. Although higher MOI (MOI = 1) had reduced the inhibition ability of *S. nigra* extract on the treated cells, viral titers were still significantly large by approximately four orders of magnitude. Virus titers decreased with increasing *S. nigra* concentrations in a dose-responsive manner [40]. 

On the other hand, the antiviral activity of *S. formosana* Nakai stem ethanol extract and some phenolic acid had a potent effect against human coronavirus HCoV-NL63 with 50% inhibitory concentration (IC_50_) of virus yield, plaque formation, and virus attachment, which were 1.17, 4.67, and 15.75 μg/mL, respectively [33]. Caffeic acid, chlorogenic acid, and gallic acid contained in *S. formosana* Nakai stem ethanol extract showed a significant reduction in HCoV-NL63 activity with IC_50_ of 3.54, 43.45, and 71.48 μM. Caffeic acid also significantly inhibited the replication and blocked the attachment of HCoV-NL63 to a host cell with IC_50_ = 8.1 μM. The study concluded that caffeic acid might be a vital component in anti-HCoV-NL63 activity. 

### 2.3. Plantago spp.

Plantago is an important medicinal plant containing different compounds, such as flavonoids, alkaloids, terpenoids, vitamin C, and phenolic compounds (caffeic acid derivatives) with powerful antioxidant properties. The Plantago spp.’s herb tea is helpful for sore throat, dry cough, and stomach irritation [41]. In southwest Iran, *P. lanceolata* L. and in Kazeroun ethnobotany, *P. coronopus* is used to treat common colds [42]. The antiviral activity of aqueous extract and pure compounds of *P. major* was examined on a series of adenoviruses (ADV-3, ADV-8, ADV-11) that commonly cause cold-like symptoms [43]. The results indicated that aqueous extract of *P. major* possessed only a slight anti-herpes virus activity. However, certain pure compounds belonging to the five different classes of chemicals found in extracts of this plant exhibited potent antiviral activity. Among them is caffeic acid that exhibited the strongest activity against ADV-3 (CC_50_ = 10,293 μg/mL, EC_50_ = 14.2 μg/mL, SI = 727), whereas chlorogenic acid possessed the strongest anti-ADV-11 (CC_50_ = 3995 μg/mL, EC_50_ = 13.3 μg/mL, SI = 301) activity. Intriguingly, the effect of chlorogenic acid (CC_50_ = 3995 μg/mL, EC_50_ = 13.3 ± 3.2 μg/mL; SI = 301) possessed similar antiviral activity but a lower cytotoxicity than the standard drug (EC_50_ = 14.0 ± 0.1 μg/mL; SI = 16.4) in inhibiting ADV-11 [43]. In addition, a study reported the in vitro antiviral activity of *P. asiatica* extract (PAE) against the respiratory syncytial virus [34].

**Figure 2:**
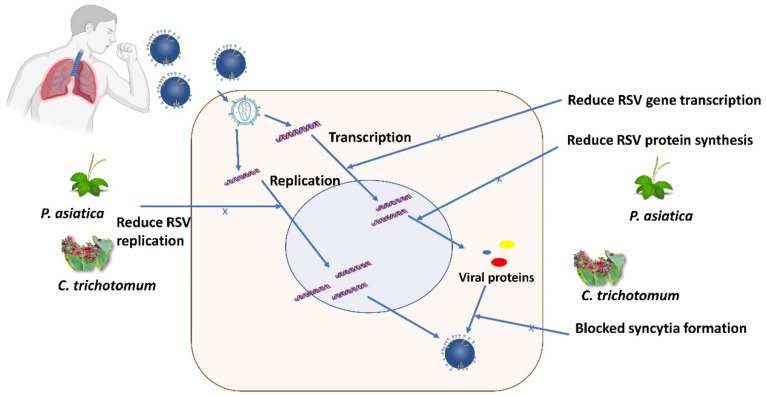
The mechanism of actions of antiviral agents from *P. asiatica* and *C. trichotomum* against the RSV. Once the virus penetrates the host cell, the life cycle of the RSV begins that can be targeted for antiviral compounds extracted from the whole plant of *P. asiatica* and *C. trichotomum.* The plant extract reduced RSV replication, RSV-induced cell death, RSV gene transcription, RSV protein synthesis, and blocked syncytia formation.

Figure 2 presents the mechanism of actions of antiviral agents from *P. asiatica* against RSV. The extractability was tested on GFP-tagged RSV on HEp2 cells and A549 cells, which was found to be dose-dependent as the extract at 50 μg/mL was the most effective at inhibiting viral replication and virus-induced cytotoxicity. For in vitro experiments, the cytotoxic concentration (CC_50_), effective concentration (EC_50_), and selectivity index (SI) of PAE were found to be 938.43, 39.82, and 23.5 μg/mL, respectively. In vivo experiments on BALB/c mice showed that PAE-treated mice significantly reduced viral mRNA in the respiratory tract at both 3 dpi and 5 dpi compared to the control phosphate-buffered saline (PBS)-treated group [34]. 

### 2.4. Tilia spp. 

*Tilia cordata* (known as small-leaved lime or linden) and *T. platyphyllus* (known as large-leaved lime or linden) are found in warmer climates in Europe. The leaves, wood, volatile oil, and charcoal of the Tilia flowers are used for medicinal purposes [44]. Both *T. plathyphyllus* and *T. cordata* are widely used traditionally as herbal tea. Commission E and British Herbal Compendium claimed that linden flowers are used for colds, any cold-related coughs, upper respiratory catarrh, common colds, irritable coughs, hypertension, and restlessness [45]. 

The flowers of *T. flos* from the family Malvaceae are taken internally as a tea to treat fever, flu, viral infections, and improved respiratory tract. The flavonoids contained in the flowers were reported to show antioxidant properties by acting as reactive oxygen species (ROS) scavengers, principally on hydrogen peroxide and the superoxide anion [46]. Czerwińska et al. (2018) evaluated the inflammatory response of procyanidins present in the flowers of *T. cordata* in human neutrophils ex vivo model. The results concluded that procyanidins were able to reduce inflammation and irritation of mucosa in common colds, pharyngitis, and tonsillitis. The investigated procyanidins (Epicatechin) possessed strong inhibition on the production of reactive oxygen species (ROS) by N-formylmethionyl-leucyl-phenylalanine (N-f-MLP) stimulated neutrophils at the lowest concentration of 1 μM [47]. 

### 2.5. Clerodendrum trichotomum

**Figure 3:**
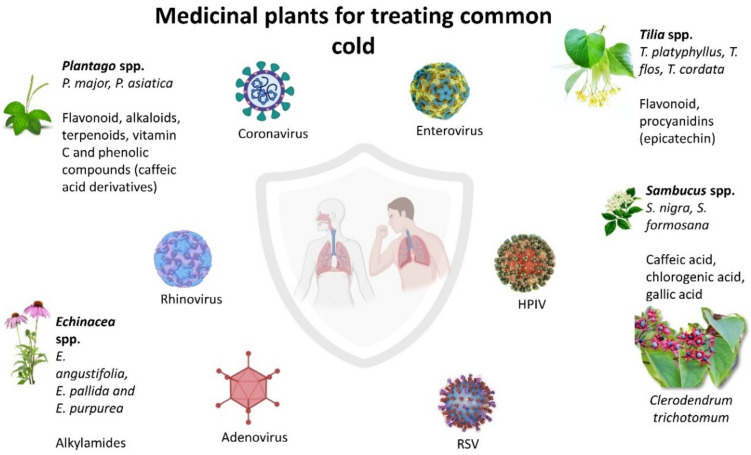
Overview of medicinal plants for treating common cold infections.

*C. trichotomum* is a plant belonging to the Lamiaceae (formerly Verbenaceae) family and grows wildly in fields and mountains in Korea, Japan, and China [48]. Chathuranga et al. (2019) studied the antiviral properties of *C. trichotomum* against the respiratory syncytial virus (RSV) in vitro cell cultures and an in vivo mouse model [34]. The treatment of HEp2 cells and A549 cells with a non-cytotoxic concentration of *C. trichotomum* extract significantly reduced RSV replication, RSV-induced cell death, RSV gene transcription, RSV protein synthesis, and blocked syncytia formation (Figure 3). Intriguingly, *C. trichotomum* extract taken orally significantly improved viral clearance due to the reduction in the RSV-G mRNA transcription (significantly reduced at 5 dpi) in the lungs of BALB/c mice. *C. trichotomum* extract significantly reduced RSV infection (MOI, 0.1) by 50% (EC_50_) at a concentration of 27.95 μg/mL, whereas the cytotoxicity concentration (CC_50_) was at 764.17 μg/mL. Both values raised the selectivity index (SI) to 27.3, indicating the safety of a crude extract against RSV infection.

## 3. Medicinal Plant for Treating Influenza

Influenza viruses consist of four distinct viruses: influenza A, B, C, and D, with influenza viruses A, B, and C commonly causing flu in humans [49]. Flu frequently comes suddenly with the abrupt onset of high-grade fever, myalgia, headache, and malaise. These situations are accompanied by symptoms of respiratory tract illnesses, such as nonproductive cough, sore throat, loss of appetite, and nasal discharge. In the worst condition, influenza can affect other organs, such as the lungs, brain, and heart, and cause hospitalization [50]. Exploration and usage of a wide range of medicinal plants for flu treatment were popular in Southeast Asia countries, such as Malaysia, Thailand, Indonesia, Vietnam, and Borneo [51,52,53]. It was reported that the extracts of fifty medicinal plants found in the tropical rainforests of Borneo had been used as herbal medicines by traditional healers to treat flu-like symptoms [53]. Table 3 recapitulates the medicinal plants possessing antiviral activity causing influenza.

### 3.1. Punica granatum

*P. granatum,* known as pomegranate, is a fruit-bearing deciduous shrub in the family Lythraceae, subfamily Punicoideae, that grows between 5 and 10 m tall. The pomegranate originated in the region, spreading from Iran to northern India, and has been cultivated throughout the Mediterranean since ancient times. The pomegranate peel’s ethanol extract and its n-butanol and ethyl acetate fractions have the highest inhibitory effect against influenza A virus, with IC_50_ values of 6.45, 6.07, and 5.6 μg/mL in MDCK cells, respectively. Upon treatment with the crude pomegranate’s polyphenolic extract (PPE) and its polar fractions in a dose-dependent manner, the production of virus significantly reduced (*p* < 0.05) [54]. Antiviral properties of pomegranate polyphenol extract (PPE) on influenza A were tested using real-time PCR, plaque assay, and TCID 50% hemagglutination assay in MDCK cells. PPE did not induce cytotoxicity in MDCK cell lines until the concentration exceeded 100 μg/mL, and concentrations of 0–40 μg/mL were used in an in vitro test. Realtime PCR test manifested the virucidal effect of PPE that inhibited the virus RNA replication in single-cycle growth conditions. The proliferation of influenza A in MDCK cells (MOI = 0.05) was blocked when MDCK cells were exposed to PPE during the viral absorption phase [55]. The potent antiviral activity of polyphenols (PP) components of pomegranates inactivated influenza virus (H1N1 and H3N2 influenza viruses and reassortant H5N1 virus rg-VN/04) through a direct effect on the viral particle [56]. Viral inactivation by PP was the result of damage to virion structural integrity, and complete influenza inactivation was shown with the treatment of 1600 μg/mL of PP. Intriguingly, even the treatment of 400 μg/mL PPs for 5 min showed a 99% virus reduction.

### 3.2. Geranium sanguineum

*G. sanguineum*, also known as bloody crane’s bill or bloody geranium, is a hardy-flowering perennial herbaceous plant of the Geraniaceae family. It is also a Northumberland County flower. A polyphenol-rich extract isolated from the aerial roots (roots exposed to the air or above the soil) of the medicinal plant *G. sanguineum* had strong anti-influenza activity [57]. Using the replication of representative influenza viruses in cell cultures, a study of cell-toxic and virus-inhibitory effects of the extract showed that the presence of various biologically active compounds as well as the possible synergistic interactions between them seemed to be decisive for the overall antiviral effect. EtOAc and *n*-BuOH extracts from aerial parts and roots of *G. sanguineum* were compared with the ethanol (EtOH) crude extract for their anti-influenza activities. Only the *n*-BuOH fraction (selectivity index, SI = 24.2) demonstrated significant in vitro antiviral activity in which out of four of the subfractions (*n*-BuOH-1, -5, -6, -7), only two of them (*n*-BuOH-5, -6) surpassed the overall effect of the total EtOH extract (SI > 50), whereas the EtOAc fraction was the most effective in an in vivo study [57]. The effect of polyphenol-rich extract of *G. sanguineum* on the proliferation of MDCK and CEF cells was determined using an MTT assay, which revealed IC_50_ values of 64 μg/mL and 72 μg/mL, respectively [58].

### 3.3. Echinacea spp.

The aqueous fraction of *Echinacea purpurea* roots was reported to show potent activity against the influenza virus. The ethyl acetate fraction *of E. angustifolia* root extract contained moderate activity against three viruses (HSV, influenza, and rhinovirus). In contrast, *E. pallida* root extracts were devoid of antiviral activity [31,68]. The minimum concentration of *E. purpurea* required to inactivate 100% virus (MIC_100_) of tablets/capsules was found to be 2.5 μg/mL. *E. purpurea* teas have a 2.2 μg/mL concentration, which clearly showed that *E. purpurea* water extract taken as tea brewed at 80 °C possessed better antiviral activity than taken as capsules/tablets. Meanwhile, among a few extracts of the root of *E. pallida* var. *angustifolia* (viz. ethanol 55% extract at 40 °C, water extract at 40 °C, water extract at 80 °C, 70% ethanol extract at 40 °C, hexane and ethyl acetate extracts), only 55% ethanol extraction at 40 °C and ethyl acetate extract showed anti-influenza activity. However, ethyl acetate extract of *E. pallida var. angustifolia* possessed the best antiviral activity against influenza with MIC_100_ = 33.5 μg/mL than ethanol extract with 348 μg/mL [37].

### 3.4. Cistus incanus

*Cistus incanus* is rich in polyphenols that include many representatives with strong antioxidant and antiviral activities [69]. A study stated that CYSTUS052, a plant extract from a special variety of *C. incanus,* rich in polymeric polyphenols exhibited antiviral activity against a highly pathogenic avian influenza A virus (FPV, H7N7) in MDCK cell culture and a mouse infection model [59]. MDCK cells were treated with 4 mL CYSTUS052 extract (1 mg/mL) as an aerosol for 20 min; after being infected through 2 mL of nebulized virus for 10 min, they showed a reduction in viral plaque numbers between 70% and 90%. Inbred female mice treated with 2 mL of aerosolized CYSTUS052 (10 mg/mL) extract for 10 min, three times a day, for 5 days and infected with FPV after the first treatment had shown no disease symptoms. Moreover, *C. incanus* extract also prevented infection of cells by virus particles containing Ebola or Marburg virus envelope proteins, together with envelope components of filoviruses [70].

### 3.5. Glycyrrhiza spp.

*Glycyrrhiza uralensis, G. inflate,* and *G. glabra* were prescribed as licorice in Chinese pharmacopeia, in which the roots and rhizomes are the main medicinal parts [71]. Glycyrrhizin, a triterpene saponin, is an active constituent of licorice root. Notably, H5N1-induced cytokine expression and H5N1-induced caspase activation were reduced. Moreover, H5N1-induced apoptosis was also inhibited by the investigated glycyrrhizin at 100 μg/mL, although H5N1 replication was not affected. In conclusion, as H5N1-induced hypercytokinaemia is considered to play an important role within H5N1 pathogenesis, glycyrrhizin may complement the arsenal of potential drugs for the treatment of H5N1 disease [61]. Studies suggested that glycyrrhizin found in *G. uralensis* was the most active compound in inhibiting the replication of SARS-associated viruses, including COVID-19 [72,73,74]. Glycyrrhizin also reduced HMGB1 binding to DNA, which is required to enhance influenza virus replication, thereby inhibiting influenza virus polymerase activity [75].

### 3.6. Chaenomeles sinensis

*Chaenomeles sinensis* is widely used as a traditional Chinese medicine to treat throat diseases [62]. The polyphenol-rich extract, CSD3, from *C. sinensis* was pre-treated for A/Udorn/72 (H3N2) virus. Pre-treatment with CSD3 mildly reduced cell-binding, hemagglutination, and hemolytic activities by 70%. The main target step of CSD3 in the replication cycle is after cell-binding but before or at primary transcription, which increases the permeability of the virus envelope. CSD3 was suggested to be employed as a lozenge or mouthwash for daily use in preventing influenza virus infections [63].

### 3.7. Sambucus nigra

*Sambucus nigra* juice (elderberry) with the addition of beta glucan 1,3/1,6, zincum gluconium, and acidum ascorbicum was studied against human pathogenic viruses: Influenza A H1N1 (FluA H1N1). *S. nigra* juice at 2.1% concentration showed a dose-dependent antiviral effect on FluA H1N1 with 2.5% concentration to exhibit the strong effect, which completely blocked the replication of the virus [76]. This juice possessed potent antiviral activity with therapeutic index of 12 ± 1.3 (CC_50_ = 770 ± 60 μg/mL on MDCK cells, CC_50_ = 810 ± 30 μg/mL on A549 cells) against influenza infection, causing flu (IC_50_ = 6000 ± 800 μg/mL). A stronger viral inhibition by elderberries was detected against the late-stage influenza cycle than in the early stage via blocking the virus glycoproteins. Elderberry acted on direct virus inhibition by suppressing viral entry, affecting the post-infection phase and viral transmission from cell to cell, together with indirect viral inhibition through modulating the release of cytokines, such as IL-6, IL-8, and TNF [64].

### 3.8. Phyllanthus emblica

1,2,3,4,6-penta-*O*-galloyl-β-D-glucose or known as PGG was isolated from the branches and leaves of *Phyllanthus emblica*. PGG was tested for its cytotoxicity on MDCK cells in a cell-based screening assay to raise its CC_50_ value of 29.59 ± 4.32l μg/mL. PGG also showed potent inhibitory activity against influenza strain A/WSN/33(H1N1) with an EC_50_ value of 2.36 ± 0.29 μg/mL and selectivity index (SI) of 12.54 [65]. According to Lv et al. (2015), phyllaemblicin B and glochicoccinoside D were identified as the main components of *P. emblica* roots and showed potential antiviral properties against H3N2 virus strain with 50% inhibitory concentration (IC_50_) of 2.6 ± 0.7 μg/mL and 4.5 ± 0.6 μg/mL, respectively [66].

### 3.9. Camellia sinensis (Green Tea)

Lee et al. (2018) evaluated the antiviral spectrum of *Camellia sinensis* (green tea) extract (GTE) using the influenza A virus/Puerto Rico/8/34 (H1N1) as a model to examine the duration of the viral inactivating activity of the GTE that was stored at various temperature conditions [77]. GTE at 0.1% (the highest concentration used) completely inactivated 10⁶ PFU (plaque forming unit) of the virus titers by 6 log_10_ reductions, and 0.01% and 0.05% concentration reduced 2 log_10_ reductions, even though it was kept in storage for more than 2 months at different temperatures (4 °C, 25 °C, and 37 °C). The addition of ascorbic acid (as an antioxidant) to GTE was found to prolong the duration of the virucidal properties of GTE. Among three polyphenolic compounds of green tea viz. (−)-epigallocatechin gallate (EGCG), (−)-epicatechin gallate (ECG), and (−)-epigallocatechin (EGC), EGCG and ECG were found to be potent inhibitors of various influenza virus subtypes (including influenza A/H1N1, A/H3N2 and B virus) replication in MDCK cell culture. The 50% effective inhibition concentration (EC_50_) of EGCG, ECG, and EGC for influenza A virus were 22–28 μM, 22–40 μM, and 309–318 μM, respectively. The study also suggested that the virucidal properties of catechin were due to the altering physical integrity of the membrane of host cells or the virus particles [67].

**Figure 4:**
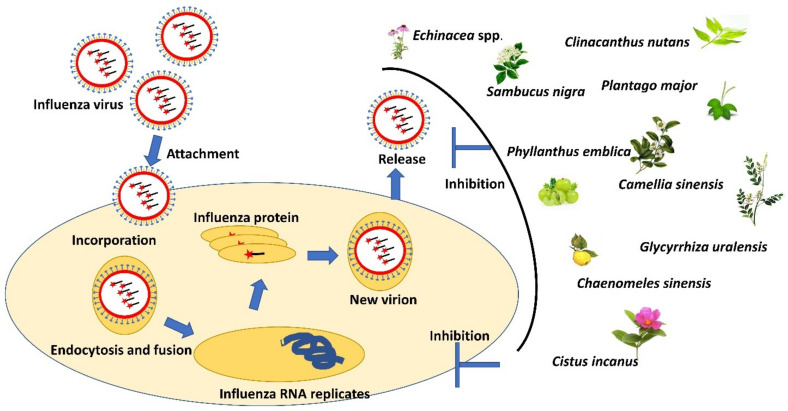
An overview of influenza mechanism of action via inhibition of viral replication and treatment using selected antiviral medicinal plants.

Figure 4 shows the overview of the influenza mechanism of action via inhibition of viral replication and treatment using selected antiviral medicinal plants where these plants may inhibit the replication and release of the influenza virus.

## 4. Medicinal Plants for Antiviral Treatment of Sexually Transmitted Diseases

Sexually transmitted diseases (STDs) by viral infections are life-threatening without a proper cure. Sexually transmitted infections (STIs) caused by viruses include hepatitis B virus (HBV), herpes simplex virus (HSV), human immunodeficiency virus (HIV) and the human papillomavirus (HPV), which contribute to adverse effects on sexual and reproductive health, including infertility in women and several different types of cancers such as cervical cancer [78]. Human papillomavirus (HPV) is the most common STD worldwide, where HPV-positive patients have a greater risk of HIV infection rather than HPV-negative patients [79]. Gheit (2019) stated that oncoproteins play a key role in inducing cervical cancer in high-risk mucosal HPV [80]. Transgender women and men who have sex with men were reported to have a high risk of HPV infection in three countries of Southeast Asia viz., Malaysia, Thailand, and Indonesia, as reported by Somia et al. (2018) [81]. Acquired Immune Deficiency Syndrome (AIDS) is a chronic and life-threatening condition caused by infections from two types of HIV: HIV-1 and HIV-2, acquired by patients through homosexual activity [82]. It was also reported that people who injected prohibited drugs showed a high prevalence of HIV infections [83]. Among two subtypes of HSV, only HSV-2 was sexually transmitted, especially among people who practice unprotected sex, such as female sex workers and men who have sex with men [84]. HSV-2 is known for causing genital mucosal ulceration and is indicated as the most prevalent sexually transmitted infection worldwide [85]. HBV can be found in blood and body fluid such as semen, vaginal fluid, and saliva. Therefore, transfusion of unscreened blood and unprotected sexual activity may spread this infection from one to another. Other than sexual transmission, HBV could cause infections via percutaneous transmission by contaminated needles and vertical transmission in utero or during delivery [78,86]. Table 4 summarizes the medicinal plants possessing antiviral activity causing sexually transmitted diseases.

### 4.1. Clinacanthus nutans and Clinacanthus siamensis

*Clinacanthus nutans* is also known as snake grass from the Acanthaceae family. This plant is popular in Malaysia, Indonesia, Thailand, and China due to its diverse and potential medicinal uses in traditional herbal medicine. According to Kunsorn et al. (2013), three extracts of the *C. nutans* and *C. siamensis* (n-hexane, dichloromethane, and methanol) leaf extracts were found to exhibit potency of antiviral activity against HSV-1 strain KOS and HSV-2 strain Baylor 186 by inhibiting viral plaque formation. The results revealed that methanolic extract of *C. nutans* exhibited only a slight anti-HSV-1 activity (IC_50_ = 64.93 µg/mL) compared to the *n*-hexane (IC_50_ = 32.05 µg/mL) and dichloromethane extract (IC_50_ = 44.50 ± 2.66 µg/mL) of *C. nutans*. In contrast, methanolic extract of *C. siamensis* exhibited the strongest activity against HSV-1 (IC_50_ = 37.39 µg/mL), while the *n*-hexane and dichloromethane extracts exhibited only a slight anti-HSV-1 activity (IC_50_ = 60.00 µg/mL and 55.69 ± 4.41 µg/mL, respectively). Contrary to the *C. siamensis*, the *n*-hexane, dichloromethane, and methanol extracts of *C. nutans* exhibited a slightly weak anti-HSV-2 activity (IC_50_ = 72.62 µg/mL, 65.19 µg/mL, 65.13 µg/mL, respectively) [87]. 

### 4.2. Cistus incanus

**Figure 5:**
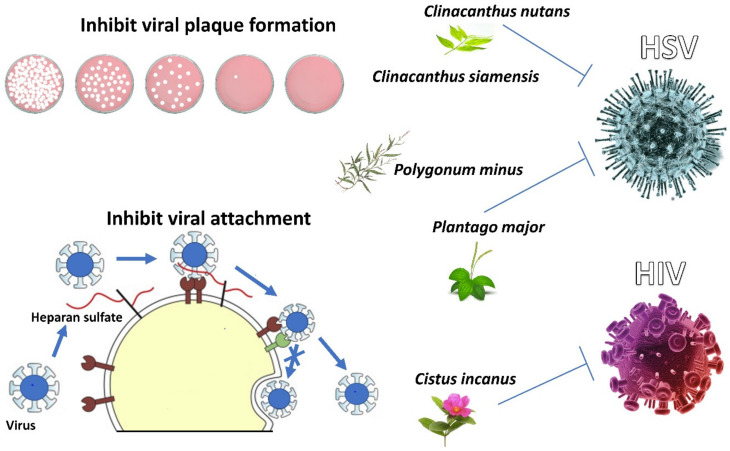
The mechanism of action of antiviral agent from *C. incanus* against HIV via inhibition of viral attachment. The mechanism of *C. nutans* and *C. siamenensis* plant extractions against HSV-1 and HSV-2 virus via inhibition viral plaque formation, whereas the mechanism of *P. minus* and *P. major* is via inhibition of viral attachment.

CYSTUS052 substance derived from the *C. incanus* extract was demonstrated to inhibit HIV-1 and HIV-2 infections in vitro (viz. EC_50_ = 8.06 μg/mL, CC_50_ = 250 μg/mL) [59,71]. Antiviral activity was highly selective for virus particles, preventing primary attachment of the virus to the cell surface and viral envelope proteins from binding to heparin. Polyphenols in *C. incanus* were attributed to its antiviral properties as a polyphenol-enriched fraction (known as CiPP) (cytotoxicity concentration, CC_50_ = 1200 μg/mL) isolated from *C. incanus* extract possessed antiviral activity when tested on HIV-1_LAI_ with lower cytotoxicity rate than *C. incanus* extract, whereas polyphenol-depleted fraction did not show any antiviral activity on HIV-1_LAI_ [59]. Figure 5 shows the mechanism of actions of antiviral agents from *C. incanus* against HIV via inhibition of viral attachment.

### 4.3. Plantago major

*Plantago major*, a popular traditional Chinese medicine, has long been used for treating various diseases varying from cold to viral hepatitis [43]. A study demonstrated that the aqueous extract of *P. major* possessed antiviral activity against HSV-2. Among the two main components found in *P. major*, caffeic acid (CC_50_ = 10,293 µg/mL) exhibited a stronger antiviral activity against HSV-1 (EC_50_ = 15.3 µg/mL, SI = 671) and HSV-2 (EC_50_ = 87.3 µg/mL, SI = 118) than chlorogenic acid (CC_50_ = 3995 µg/mL) against HSV-1 (EC_50_ = 47.6 µg/mL, SI = 83.9) and HSV-2 (EC_50_ = 86.5 µg/mL, SI = 46.2) [43].

### 4.4. Polygonum minus

*Polygonum minus Huds* (Polygonaceae), commonly known as kesum or *Persicaria minor,* is considered one of the most important aromatic plants in Southeast Asia. The cytotoxicity and antiviral properties of methanolic extracts from the leaves and stem of *P. minus* were investigated. The LC_50_ value for leaf extract towards Vero cells was 875 µg/mL, while the LC_50_ value for stem extract was 95 µg/mL. Antiviral tests were performed on Vero cells infected with HSV-1 at three different concentrations of extract: 1.0 LC_50_, 0.1 LC_50_, and 0.01 LC_50_. The result showed that the treatment of cells with stem extract at the concentrations corresponding to 0.1 LC_50_ gave a higher cell survival compared to other concentrations [88]. This indicates that the extract can protect cells against viral attachment.

Figure 5 captures the mechanism of action from *C. incanus* against HIV via inhibition of viral attachment. However, the mechanism of action from *C. nutans* and *C. siamenensis* plant extractions against HSV-1 and HSV-2 virus is via inhibition of viral plaque formation, whereas the mechanism of *P.minus* and *P.major* is via inhibition of viral attachment.

## 5. Medicinal Plant for Treating Dermatology-Related Viruses

Cutaneous Human papillomavirus (HPV), mainly from beta and gamma genera, is widely present on the surface of the skin and transmitted by skin-to-skin contact and enters the body via cutaneous layers [89]. These so-called cutaneous HPV types induce asymptomatic chronic infections and can induce benign skin lesions, cutaneous papillomas, or warts, whereas beta HPV types appear to play a role in the initiation of skin carcinogenesis [80]. Varicella-zoster virus (VZV) is a type of human neurotropic alpha-herpesvirus. Primary infection of the virus causes varicella (chickenpox), which only naturally infects humans, with no animal reservoir. Positive infections and transmission from one patient to another are usually detected by the presence of skin lesions, rash, and meningitis [90,91]. Enterovirus A71 (EV-A71) and coxsackievirus A6 (CV-A6) are enteroviruses from the family of Picornaviridae and are the major causes of hand, foot, and mouth disease (HFMD), a major public health issue in Asia and has global pandemic potential. HFMD is an emerging infection that has overwhelmed countries in the Asia–Pacific region over the past 2 decades. Enterovirus A71 (EV-A71) and coxsackievirus A6 (CV-A6) are transmitted from person-to-person transmission by fecal–oral or oral–oral routes [92]. Other than that, herpes simplex virus (HSV), coxsackie virus, and Epstein–Barr virus may also trigger erythema multiforme, a skin condition resulting from a cell-mediated immune reaction against viral antigen-positive cells that contain the virus DNA polymerase gene [93,94]. Traditional medicinal resources were found to play an important role in the management of skin disorders, especially plants. They were used in many countries around the world in the treatment of skin ailments, where they make a major contribution to the primary health care of the population. In South Africa, most people still depend largely on medicinal plants to treat skin disorders, especially among rural communities [95]. Table 5summarizes the medicinal plants possessing antiviral activity causing dermatology-related diseases.

### 5.1. Sarracenia purpurea

*Sarracenia purpurea* (Pitcher plant), often referred to as the purple-colored pitcher plant, is a carnivorous plant of the family Sarraceniaceae. It is mainly found in the USA and Canada [71]. Antipox virus activity of *S. purpurea* on the causative agent of smallpox was pointed out by Arndt et al. (2012), where *S. purpurea* treatment worked on preventing early vaccinia virus protein synthesis and replication of monkeypox virus and variola virus [96]. The dose that induced EC_50_ of *S. purpurea* against poxvirus was 10–15 µg/mL, while the dose that induced 50% cytotoxicity (CC_50_) was 70–75 µg/mL, resulting in an SI of approximately 5–7. According to a small pilot clinical trial conducted by Dah et al. (2017), the application of an aqueous extract of *S. purpurea* in gel base alleviates symptoms, supports healing, and improves the appearance of cold sores caused by herpes simplex virus [109]. Kannan et al. (2020) demonstrated that *S. purpurea* extracts are able to hinder the replication of HSV-1 by two mechanisms of action, which are through (1) inhibiting extracellular virions or viral attachment to the human host cell and (2) inhibiting the expression of viral immediate–early, early, and late genes when added at various times post-infection. This botanical has also previously been shown to inhibit the replication of poxviruses via the early inhibition of viral gene transcription [110].

### 5.2. Clinacanthus nutans (Lindau)

The whole plant of *C. nutans* possesses anti-inflammatory properties that are superior to benzydamine for the prevention of oral mucositis, which can be worsened with the presence of the herpes simplex virus [111,112]. A single-blinded randomized clinical trial administered glycerin papayor extract of *C. nutans* orally as well as applied the extract on the lesion of oral mucositis of 60 patients. Pain severity of the patients was less on glycerin papayor extract treatments with later onset of oral mucositis than benzydamine used in the controlled group [97,111]. A clinical trial was conducted to evaluate *C. nutans’* anti-VZV activity. In their clinical study, the *C. nutans* extract was formulated into a 5% *C. nutans* cream prior to testing its ability to combat VZV infections, where the organic extract exhibited anti-VZV activity through the direct inactivation stage and a positive curing effect [98].

### 5.3. Matricaria recutita

*Matricaria recutita* (German chamomile) is one of the commonly used medicinal plants. Its flower is externally used for skin inflammations and irritations, bacterial skin diseases, nappy rash and cradle cap, eczema, poorly healed infected wounds, abscesses, frostbite, and insect bites. Moreover, its flower is also used for baths, compresses, or rinses and poultices [113]. The essential oil from *Matricaria recutita* consists of some of the largest group of medically important compounds (viz. chamazulene, epi-α-bisabolol, α-bisabolol oxide, carvacrol, para-cymene, (E)-β-ocimene, (Z)-β-ocimene, (E,E)-farnesol, and en-yn-dicycloethers) together with 0.75% of volatile oil [114]. The essential oil of chamomile has shown to be a promising antiviral agent against herpes simplex virus type 2 (HSV-2) in vitro using RC-37 cells through a plaque reduction assay, which showed a 50% inhibitory concentration (IC_50_) of 0.003% towards the virus [99].

### 5.4. Aloe vera

*Aloe vera* (Aloe) is an important and traditional medicinal plant belonging to the family Liliaceae, and it is indigenous to Africa and Mediterranean countries. The topical application of *A. vera* helps to prevent herpes ulcers and enhances the healing process of dermal injuries [115]. *A. vera* gel is recognized as antiseptic as it was reported to kill or control mold, bacteria, fungus, and viruses with the ability to eliminate internal and external infections [116]. Rezazadeh et al. (2016) measured the assessment of anti-HSV-1 activity of *A. vera* gel extract, whereby it showed a significant inhibitory effect of 0.2–5% on HSV-1 growth in the Vero cell line [100]. Higher concentrations of *A. vera* gel (1, 2, and 5%) exhibited even a more significantly anti-viral activity than 0.2% and 0.5% concentrations (*p* < 0.05), while 5% concentration had the maximum effect in reducing virus plaques presented after virus infection in the cells. A five percent concentration of *A. vera* gel extract was found to be nontoxic when tested on the Vero cell line. Zandi et al. (2007) tested the antiviral activity of a crude hot glycerin extract of *A. vera* gel against HSV-2 replication in the Vero cell line. The extract showed antiviral activity against HSV-2 before attachment, entry of virus to the Vero cells (IC_50_ = 428 µg/mL, SI value = 7.56) and post attachment stages of virus replication (IC_50_ = 536 µg/mL, SI value = 6.04) with CC_50_ = 3238 µg/mL. It was suggested that *A. vera* could be a good candidate as a natural source for antiviral drug development against HSV-2 [101].

### 5.5. Cornus spp.

*Cornus officinalis* (Asiatic dogwood, cornel dogwood) is a deciduous tree found mostly in China as well as in Korea and Japan. Other common names for this plant are Japanese cornelian cherry and Japanese cornel. The fruits of this plant have been used in all areas of its geographical distribution for a wide variety of diseases and complaints. The effect on CVA16 (Coxsackievirus A16, a serotype of the genus Enterovirus of the Picornaviridae family and one of the causative agents of hand, foot, and mouth disease (HFMD)) induced a cytopathic effect CPE, which was observed in the *C. officinalis*. The plant extract had shown significant cell viability of >50%, indicating antiviral activity against CVA16 [102]. Meanwhile, according to Lavoie et al. (2017), *C. canadensis* L. or known as bunchberry dogwood (from the family *Cornaceae*), is a plant used in Native American traditional medicine to treat possible antiviral infections [117]. The extracts were tested on herpes simplex virus type-1 (HSV-1), using a plaque reduction assay with water/ethanol 1:1 infusion of *C. canadensis* leaves, which was the most active extract that was able to inhibit virus absorption with EC_50_ of about 9 μg/mL [118].

### 5.6. Lysimachia mauritiana

*Lysimachia mauritania* is a biennial herb distributed worldwide, especially in the temperate and subtropical climate regions of both hemispheres, along coastal regions in East Asia, the Philippines, Micronesia, Polynesia, and the Indian Ocean islands. The data showed that *L. mauritiana* reduced lytic gene expression of the varicella-zoster virus and inhibited replication of the varicella-zoster virus (VZV). *L. mauritiana* mediated substantial downregulation of IE62 protein and major transactivation of IE, E, and L genes, which in turn resulted in the repression of E and L genes and inhibition of replication of the varicella-zoster virus. It was suggested that *L. mauritiana* might provide an important basis for the further development and tailoring of novel therapeutic agents to treat virus-associated diseases of varicella-zoster. The 50% inhibitory concentration (IC_50_) of *L. mauritania* extract for VZV was calculated as 26.09 µg/mL [103].

### 5.7. Mentha haplocalyx

*Mentha haplocalyx* is one of the most popular medicinal plants in the Lamiaceae family that is widely used in Chinese herbal medicine to treat various disorders and is also used as a supplement. *M. haplocalyx* was demonstrated to exert antiviral activity in blocking viral infection proinflammatory response. Its water extract was active against coxsackie A16 virus, the causative agent of hand, foot, and mouth disease (HMFD), with a 50% inhibitory concentration (IC_50_) value of 70.3 μg/mL. It significantly blocked the cytopathic effect of the coxsackie A16 virus [104]. In addition, there is a claim that mentioned various parts of the same plant were used to treat sores and rashes on the skin and mouth ulcers [119].

### 5.8. Camellia sinensis

*C. sinensis* (green tea) leaf contains large quantities of flavonoids called catechins, such as epigallocatechin and epigallocatechin gallate (EGCG). These polyphenolic compounds of green tea were reported to possess many health benefits, focusing specifically on the anti-HPV (human papillomavirus) effects of green tea, particularly after catechins were approved by the Food and Drug Administration (FDA) in 2006. The mechanism of action of green tea catechins against Human papillomavirus (HPV) is unclear; however, based on comprehensive clinical studies, they are unmistakably successful [120]. Polyphenon E (Poly E) (a well-defined pharmaceutical-grade decaffeinated green tea catechin mixture, including epicatechin (EC), epigallocatechin (EGC), epicatechin gallate (ECG), and most abundantly, approximately 65% of EGCG and (−)-epigallocatechin gallate (EGCG) were tested at different concentrations (0, 1, 5, 10, 25, and 50 µg/mL) to investigate the effect on the growth of HPV-immortalized cervical epithelial (TCL-1) cells. At lower concentrations (1 and 5 µg/mL), EGCG and poly E induced a similar level of cell growth inhibition (average inhibition rates were 19.2% and 33.4% after EGCG treatment and 25.9% and 33.8% after poly E treatment). At high concentrations (10, 25, and 50 µg/mL), EGCG caused a stronger growth inhibitory effect than poly E (72.7, 96.6, and 94.2% after EGCG treatment and 62.9, 69.6, and 83.9% after poly E treatment, respectively). Apoptosis was induced in TCL-1 cells significantly in a single dose of 50 µg/mL poly E than a single dose of 50 µg/mL EGCG. The expression of HPV-E7 in TCL-1 treated with 0 or 50 µg/mL EGCG or poly E was stained by immunohistochemistry. Untreated TCL-1 showed strong positive staining of HPV-E7, whereas both EGCG and poly E-treated groups showed a decrease in HPV-E7 expression [105].

### 5.9. Hibiscuss sabdariffa

*Hibiscus sabdariffa* is a shrub belonging to the family Malvaceae. It is a popular vegetable in Indonesia, India, West Africa, and many tropical regions and it is well known as one of the ethnomedicinal plants in Nigeria [121]. Sunday et al. (2010) aimed to study the *H. sabdariffa* leaves (red and green leaves) antiviral activities against measles virus (MV) as well as the effects of the extracts on Hep-2 cells [106]. The pre-inoculative treatment of Hep-2 cells with plant extracts showed that *H. sabdariffa* had antiviral activities only at 10 and 15 mg/mL against MV. The result of this research suggested the promising antiviral activity of *H. sabdariffa* plant extracts.

### 5.10. Glycyrrhiza glabra and Glycyrrhiza uralensis

Extract of *G. glabra* roots or known as Licorice roots, is often used in ancient Siddha medicine and is approved by the German commission [122]. Tanemoto et al. (2015) stated in the Japanese Pharmacopoeia that licorices are defined as dried roots, and stolons of *G. uralensis* Fischer and *G. glabra* L. are designated as *G. radix* (*kanzo* in Japanese) [123]. A study validated the medicinal usefulness of radices *G. uralensis* against the etiological agents of HFMD through the identification of glycyrrhizic acid (GA) as the antiviral component of *G. uralensis* against enterovirus 71 (EV71) and coxsackievirus (CVA16) infection [107]. In addition, it was also revealed that GA inhibited EV71 and CVA16 with distinct mechanisms. Another study of water extract of *G. uralensis* possessed anti-EV71 infection at IC_50_ = 0.056 μg/mL [108]. A study showed the inhibitory activity of licorice crude powder extract containing 125 µg/mL glycyrrhizin against isolated varicella-zoster virus (VZV). However, the crude form of glycyrrhizin had low antiviral activity against VZV compared to acyclovir and interferon [124].

### 5.11. Phyllanthus spp.

Glochicoccinoside D and phyllaemblicin C were identified as the main components of *Phyllanthus emblica* that possess antiviral properties against the EV71 virus strain that mainly causes hand, foot, and mouth disease (HFMD) with IC_50_ values of 2.6 ± 0.7 and 2.6 ± 0.8 μg/mL, respectively [66]. Sarisetyaningtyas et al. (2006) carried out a double-blind, randomized controlled trial on 2–14 years old subjects who experienced varicella to test whether *P. niruri* Linn. could suppress the complications after the extract syrup was taken orally, three times per day for 5 days. Forty-six subjects (51.1%) had no new papules occur after 5 days of oral administration of *P. niruri* extract syrup [125].

**Figure 6:**
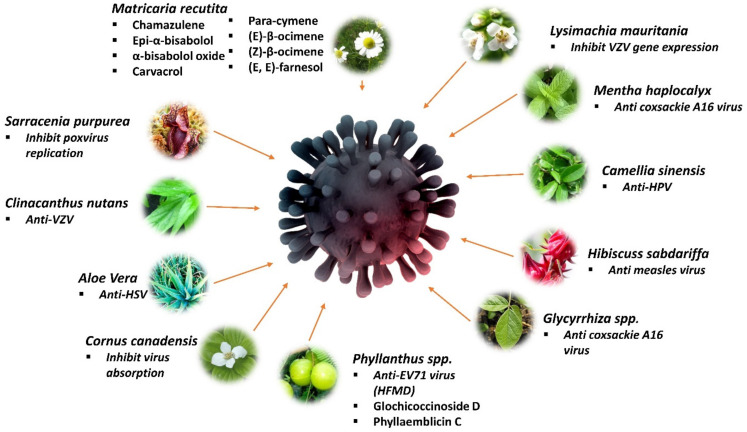
Mechanisms and compounds of medicinal plants that have antiviral activities against dermatology-related viruses.

Figure 6 summarizes the mechanisms and compounds of medicinal plants, including *Matria recutita*, *Lysimachia mauritania*, *Menta haplocalyx*, *Camellia sinensis*, *Hibiscuss sabdariffa*, *Glycyrrhiza* spp., *Phyllantus* spp., *Cornus canadensis*, *Aloe barbadensis*, *Clinacanthus nutans*, and *Sarracenia purpurea* that have antiviral activities against dermatology related viruses.

## 6. Medicinal Plants for Treating Mosquito-Borne Viral Disease

Mosquito-borne viral diseases can be transmitted to humans and animals by viral-infected mosquitoes. Some of the common mosquito-borne viral diseases are dengue, Zika, Chikungunya fever, yellow fever, West Nile virus, eastern equine encephalitis, western equine encephalitis, St. Louis encephalitis, and La Crosse. Only certain types of mosquitoes can carry viruses, and very few are even infected by feeding on an infected host. Mosquito populations are the highest from spring to fall, causing a spike in mosquito-borne viral diseases or any mosquito-borne disease. Children below 12 and elderlies over 50 years old have higher chances of experiencing severe symptoms. The disease onset may be fever, headache, muscle aches, nausea, or vomiting, and may progress to seizures, paralysis, coma, and possibly death. Preventing mosquito bites is the most effective way of preventing the spread of these diseases [126]. Table 6 shows the medicinal plants possessing antiviral activity against mosquito-borne viral disease.

### 6.1. Anti-Dengue Medicinal Plants

Dengue fever, with its severe manifestations in the form of Dengue Haemorrhagic Fever (DHF) and Dengue Shock Syndrome (DSS), has emerged as a great public health concern, spreading to all tropical and sub-tropical countries in the world, particularly important in Southeast Asia, which bear a high burden of dengue. The dengue virus (DEN virus) is a member of the family Flaviviridae with four antigenically related but distinct serotypes: DENV-1, DENV-2, DENV-3, and DENV-4. The incidence of disease and its severity varies between primary and secondary infections, possibly also across different dengue virus serotypes [140]. Ooi and Gubler (2009) stated that dengue fever in Southeast Asia emerged during and after the Second World War, whereby the movement of equipment and people helped in the spread of the infections into new geographic areas [141].

#### 6.1.1. *Alternanthera philoxeroids*

An immersed aquatic plant, *A. philoxeroids* or also called “Alligators Weed’’, belongs to the family Amaranthaceae, which originated from South America but is currently invading Australia. Jiang et al. (2005) investigated an in vitro effect of *A. philoxeroides* extract against the dengue virus. An MTT assay was initially carried out to determine the cytotoxicity of *A. philoxeroides* extract on C6/36 cell lines. Coumarin-based extract of *A. philoxeroides* showed the lowest toxicity on cells (TD_50_ = 535.91), whereas a petroleum ether extract of *A. philoxeroides* had the strongest inhibitory effect on dengue virus (ED_50_ = 47.43) [127].

#### 6.1.2. *Cladosiphon okamuranus*

*Cladosiphon okamuranus* belongs to the family Chordariaceae. It is a type of brown seaweed found naturally in Okinawa, Japan. Hidari et al. (2008) stated that a sulfated polysaccharide named fucoidan from *C. okamuranus* was found to potentially inhibit DENV-2 infection [128,142]. The infection of the virus was tested in BHK-21 cells. Fucoidan reduced infectivity by 20% at 10 μg/mL compared to untreated cells.

#### 6.1.3. *Cladogynos orientalis*

*Cladogynos orientalis* belongs to the family Euphorbiaceae. This white stellate-hairy shrub is about 2 m high and is found in Southeast Asia, Malaysia, and Thailand. The dichloromethane ethanol extract of *C. orientalis* was tested in vitro for an anti-dengue activity against DENV-2 in Vero cells through the MTT assay. The results showed that the ethanol extract of *C. orientalis* at a concentration of 12.5 μg/mL exhibited 34.85% inhibitory activity against DENV-2. In addition, *C. orientalis* at a concentration of 100 μg/mL also exhibited an inactivated viral particle activity at about 2.9% [143].

#### 6.1.4. *Clinacanthus nutans*

The 80% ethanol extract of the aerial part of *C. nutans* demonstrated a moderate anti-dengue virus activity with an IC_50_ value of 31.04 μg/mL [129]. The pheophorbide A isolated from the chloroform extract of *C. nutans* was able to inhibit dengue viral 2 replication in the direct inactivation and post-incubation stages with a CC_50_ of 25 μg/mL [130]. Other extract and plant parts can be tested on this virus to evaluate its effectiveness.

#### 6.1.5. *Sambucus nigra*

The antiviral activity of the methanolic extracts (leaves and flowers) of *S. nigra* was tested on dengue virus serotype-2 (DENV-2). A study showed that 400 µg/mL methanolic extracts of both plant parts possessed a protective effect against DENV-2 and an effective method to protect cell monolayers when the cells were treated with the extracts before they were infected [132]. This indicates that pre-treatment with *S. nigra* flowers extract was more protective than pre-treatment with *S. nigra* leaf extract.

#### 6.1.6. *Carica papaya*

*Carica papaya* (papaya) is a popular and important fruit in tropical and subtropical regions, which is consumed worldwide. Thirty percent of the worldwide production of papaya is from Asia. Papaya is a family member of Caricaceae that is rich in vitamin A, vitamin C, and vitamin E and possesses antiviral, antibacterial, and antifungal properties [144]. A study about an in vitro test of chloroform extract of *C. papaya*’s leaves showed that it has a moderate inhibitory action against the dengue virus growth on LLC-MK2 cell line with EC_50_ of 1000 μg/mL and selective index of 1 [133]. The chloroform extract of *C. papaya*’s leaves was also non-cytotoxic on LLC-MK2 cells (CC_50_ = 1000 μg/mL). Another study stated that aqueous extract of leaves from *C. papaya* had a promising anti-dengue activity on Vero cells infected with dengue virus 2 (CC_50_ = 10437 μg/mL, IC_50_ = 137.6 µg/mL, SI value = 75.85) [134]. The antiviral activity was evaluated by the capability of aqueous extract of leaves from *C. papaya* to significantly decrease dengue viral foci. An open-labeled randomized controlled trial was conducted on 288 patients with dengue fever and dengue hemorrhagic fever aged less than 60 years old to investigate the platelet-increasing property of *C. papaya* leaves juice. Platelet count of dengue patients significantly increased after 40–48 h of *C. papaya* leaves juice consumption during dengue infection [145]. The platelet counts of a 45 years old dengue patient were increased when treated with 25 mL of aqueous extract of *C. papaya* twice per day for five days [146].

### 6.2. Anti-Chikungunya Medicinal Plants

Another mosquito-borne disease, Chikungunya virus (CHIKV), developed from a relatively obscure and geographically isolated pathogen into a serious public health hazard in recent years. It has now been discovered in Africa, Asia, Central, and South America, with the potential to spread to North America. *Aedes aegypti* and *A. albopictus* are the most common vectors of the chikungunya virus (CHIKV). CHIKV can be transmitted to a new, naïve host more quickly than for other mosquito-borne viruses as the viruses can be found in the saliva of the mosquitos. The viruses replicate in the midgut, disseminate into salivary glands, and can be found in saliva 2–3 days after the blood meal [147]. CHIKV infection causes fever and severe joint pain. Other symptoms include muscle pain, joint swelling, headache, nausea, fatigue, and rash [148]. Various traditional medicinal plants have been reported to exert antiviral effects against CHIKV.

#### 6.2.1. *Picrorhiza kurroa*, *Ocimum tenuiflorum*, and *Terminalia chebula*

The aqueous extracts of three plants, including *Picrorhiza kurroa* (rhizome part), *Ocimum tenuiflorum* (whole plant), and *Terminalia chebula* (fruit), showed anti-CHIKV activity by inhibiting viral attachment as well as replication inhibition by inhibiting helicase and protease activities [135]. All three plant extracts with a standard concentration of 10 μg/mL (minimum concentration to inhibit the plaque without causing cytotoxicity in the drug alone) inhibited 80% of CHIKV plaque formation. When the Vero cells infected with a fixed concentration of CHIKV for 2 h were treated with 25, 50, 100, or 200 μg/mL of *O. tenuiflorum* and *T. chebula* aqueous extracts, the number of plaques also decreased as the extract concentration increased. Complete plaque inhibition (100% inhibition) was observed at 200 μg/mL. Meanwhile, *P. kurroa* aqueous extract showed only 67% inhibition at 200 μg/mL. 

#### 6.2.2. *Rhapis excelsa* and *Vernonia amygdalina*

Chan et al. (2016) revealed that *Rhapis excelsa* (family Arecaceae) and *Vernonia amygdalina* (family Compositae) could be the sources of anti-Chikungunya virus agents [136]. Both plant extracts showed strong cytopathic effect inhibitory activity against the Chikungunya virus on African monkey kidney epithelial (Vero) cells. The quantitative RT-PCR analysis on the chloroform extract of leaves from *R. excelsa* resulted in the highest percentage of reduction in the viral load (98.1%), followed by the ethyl acetate extract of leaves of *V. amygdalina* (95.5%). In addition, the chloroform extract of *R. excelsa* and the ethyl acetate extract of *V. amygdalina* had the lowest EC_50_ values of 29.9 ± 0.9 and 32.4 ± 1.3 μg/mL, respectively. Cytotoxicity assay showed the CC_50_ values of each plant extracts of 161.5 ± 19.2 and 165.5 ± 9.2 μg/mL, respectively.

#### 6.2.3. *Nicotiana benthamiana*

The viability of using an anti-CHIKV monoclonal antibody (mAb) expressed and assembled in wild-type and glycoengineered *Nicotiana benthamiana* in treating CHIKV infection in vivo and in vitro [137]. CHKVmAbs were efficiently extracted from leaves of two types of *N. benthamiana* (wild-type and glycoengineered-type of *N. benthamiana*) with the potential of neutralizing CHIKV in vitro and showed efficacy but with different potency against CHIKV infection in a murine model. The plaque reduction neutralization test (PRNT) was used to show strong neutralization activities of both mAb variants against CHIKV with statistically similar potency and EC_50_ of 130.5 μg/mL and 390.8 μg/mL for glycoengineered and wild type, respectively. An in vitro test of mAb from both types of *N. benthamiana* on five weeks old C57BL/6 mice showed a significantly lower viremia compared to PBS-treated mice.

### 6.3. Anti-Zika Medicinal Plants

Zika virus (ZIKV) is spread by mosquitoes, which is a mosquitoes-borne RNA virus of the Flavivirus genus that could cause congenital microcephaly and hemorrhage. This infection became a big global issue and a worldwide epidemic, which raised a huge concern. As the virus emerged, many tried to identify the plants that could be used as an alternative medicine against the Zika virus. Research studies showed positive results, which proved that medical plant-based extracts could be useful in treating the virus [149].

#### 6.3.1. *Aphloia theiformis*

*Aphloia theiformis* is a slender, evergreen tree with drooping branches, growing up to 20 m tall. This plant is well distributed around Reunion Island, a French department in the Indian Ocean. An analysis *of A. theiformis* showed its richness in phenolic compounds, mainly C-glycosylated xanthones, such as mangiferin and flavonoids, that possess many beneficial properties, including antiviral activities. Such antiviral properties are played by polyphenols, such as delphinidin, baicalein, and naringin, that were reported to inhibit the early steps of ZIKV replication [138]. The extract and components of *A. theiformis* were stable, and the antiviral properties were shown by interfering with at least one of the early steps of ZIKV infection. The result revealed that it took 10–15 min to inhibit the Green Fluorescent Protein (GFP) expression by 50% at non-cytotoxic concentrations of *A. theiformis* extract [138]. *A. theiformis* represented a high potential for prophylactic agents, targeting the entry of two medically relevant flaviviruses and could be used to treat patients. The concentrations of *A. theiformis* extract that inhibited 50% of cell viability (CC_50_) was at 3000 µg/mL, whereas the concentration that inhibited 50% of viral infectivity (IC_50_) was at 100 µg/mL, resulting in the selectivity index of *A. theiformis* extract of 30.

#### 6.3.2. *Nicotiana benthamiana*

*N. benthamiana* is a plant producing ZIKV E (zE) that correlated with protective immunity against multiple ZIKV strains [150]. The developed ZIKV E (zE) (Envelope (E)), a zE-based subunit vaccine, was produced via a transient expression as it is more potent at inducing strong neutralizing antibody and cellular immune responses. The protein-based plant-produced zE (PzE) is potentially safe as it eliminates the risk of genome insertion and oncogenesis of DNA vaccines. Purified PzE from the leaves of *N. benthamiana* using SDS-PAGE analysis, Ni^2+^ affinity chromatography was effective in removing *N. benthamiana* host proteins and was able to enrich PzE to >90% purity. Yang et al. (2018) also stated that the treatment of C57BL/6 mice with PzE through injection had elicited a potent humoral response, which exceeded the required threshold that correlated with the protective immunity against ZIKV [150]. This suggested that the PzE immunization regime has a good potency in eliciting IgG response against ZIKV and may also protect mice from lethal ZIKV challenges in the in vivo study.

#### 6.3.3. *Psiloxylon mauritianum*

Clain et al. (2019) demonstrated the inhibition of different ZIKV African and Asian strains infection in vitro by *Psiloxylon mauritianum* fresh aerial aqueous extract [139]. Cytotoxicity effects of *P. mauritianum* extract were performed on mammalian cell lines using MTT assay, which resulted in the extract not exhibiting cytotoxic effects on Vero, A549 cells, human primary keratinocytes (HKPM), and fibroblast (FMa) cells with CC_50_ 1044 ± 106.2 μg/mL, 657 ± 15.7 μg/mL, 353 ± 84.4 μg/mL, and 820 ± 26.5 μg/mL, respectively. Genotoxic effects of *P. mauritianum* were determined using COMET assay on mammalian cell lines and did not cause any damaging effects on the mammalian cell DNA. A viral inactivation assay was performed to determine if the *P. mauritianum* extract had the ability to neutralize ZIKV infectivity. The concentration of *P. mauritianum* that inhibited 50% of ZIKV infection (IC_50_) was estimated at 19.5 ± 4.8 μg/mL with a selectivity index calculated as 53.5. The time-of-drug-addition assays revealed that the *P. mauritianum* extract interfered with the attachment of the viral particles to the host cells.

**Figure 7:**
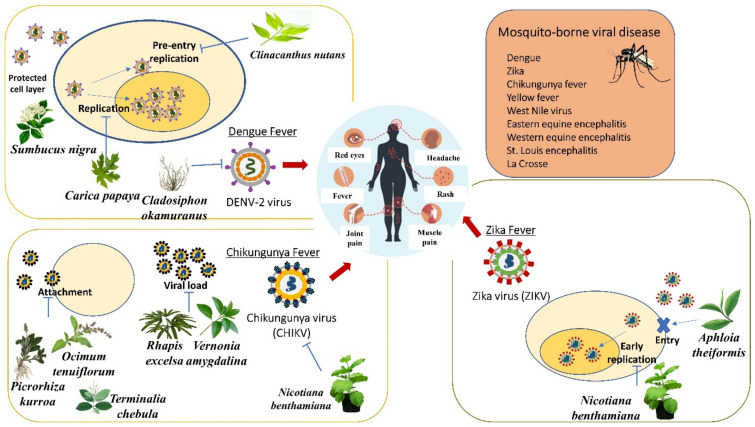
Overview of medicinal plants for treating mosquito-borne virus. *C. nutans, S. nigra, C. papaya* inhibited the replication of DENV-2 virus. *O. tenuiflorum, P. kurroa,* and *T. chebula* inhibited the viral attachment. *R. excelsa* and *V. amygdalina* inhibited the viral load of Chikungunya virus. *N. benthamiana* inhibited the early replication, whereas *A. theiformis* inhibited the entry of Zika virus.

Figure 7 recapitulates the overview of medicinal plants for treating the mosquito-borne virus. *Sumbucus nigra*, *Clinicanthus nutans*, *Carica papaya,* and *Cladosiphon okamuranus* inhibited the replication of the DENV-2 virus. *Ocimum tenuiflorum, Picrorhiza kurroa,* and *Terminalia chebula* inhibited the viral attachment. Moreover, *Rhapis excelsa* and *Vernonia amygdalina* inhibited the viral load of the Chikungunya virus. *Nicotiana benthamiana* inhibited the early replication, whereas *Aphloia theiformis* inhibited the entry of the Zika virus.

## 7. Discussion

Humankind has dealt with various diseases for centuries. This brought the urge to utilize drugs from various parts of medicinal plants. Abundant sources, such as documents, preserved monuments, and original medicinal plants, are shown as evidence in the search for drugs from nature [4]. Ancient systems of medicine such as Ayurvedic, Unani, and Traditional Chinese Medicines had shown solid evidence of medicinal plants utilized for the treatment of diseases, restoring and balancing body systems [111]. Ancient Egyptian physicians made use of nature, especially medicinal plants, and basic knowledge of human anatomy to treat many diseases manifested effectively during that era [151]. Medicinal plants and plant-based medicines also had been increasingly used in modern treatment as an alternative to synthetic medicines.

Among all plants that were reviewed, 11 plants appeared to be the most promising plants to fight against viral infections. *Sambucus nigra* and *Clinacanthus nutans* emerged as the most antiviral medicinal plants mentioned in this review against three viral infections. *S. nigra* significantly showed antiviral properties against common colds, influenza, and mosquito-borne infections, whereas *C. nutans* is effective against sexually transmitted diseases, dermatology, and mosquito-borne infections. *Echinacea purpurea, Echinacea augustofolia, and Echinacea pallida* are effective against common cold and influenza virus infections. *Plantago major* was reported to be effective against common colds and STDs. *Glycyrrhiza uralensis*, *Phyllanthus emblica*, *Camellia sinensis,* and *Cistus incanus* are effective against influenza and dermatology-related infections. *Nicotiana benthamiana* is effective against two types of mosquito-borne viral infections, which are Chikungunya and Zika viruses.

*S. nigra* fruits contain antiviral activity against infective bronchitis virus at an early point of infection and influenza A virus, while the leaves and flowers have antiviral properties against dengue virus type 2 by protecting monolayers cells and preventing viral infections [40,76,132]. In addition, Sambucus juice of the berries showed an antiviral effect on FluA H1N1 and, in combination with Pelargonium extract, had enhanced the antiviral effect against the respiratory syncytial virus [50]. Phenolic acids, flavonoids, catechins, and pro-anthocyanidins obtained from Sambucus plants showed antiviral properties on human and animal viruses, such as influenza viruses, human immunodeficiency virus, dengue virus, human herpesvirus type 1, and human coronavirus NL63 [152]. Sambucus or elderberry has inhibitory effects against influenza infection in the early stage and post-infection stage of influenza infection. A low concentration of elderberry juice (high dilution) has a greater anti-influenza effect on the post-infection stage than in the early stage. The same concentration of elderberry also showed antimicrobial activity against Gram-positive (*Streptococcus pyogenes* and group C and G *Streptococci*) and Gram-negative bacteria (*Branhamella catarrhalis*) in liquid cultures. Intriguingly, elderberry does promote an indirect viral immune response in the infected body, whereas cyanidin 3-glucoside, a major bioactive compound, only promotes a direct response to the influenza infection [64].

*Clinacanthus nutans* are known as snake grass belonging to the Acanthaceae family. This plant has diverse and potential medicinal uses in traditional herbal medicine for treating skin rashes, insects and snake bites, lesions caused by herpes simplex viral lesion, diabetes, as well as gout, where it is widely used in Malaysia, Indonesia, Thailand, and China [153]. According to Thongchai et al. (2010), crude ethyl acetate extract of *C.* nutans’s leaves possesses anti-HIV-1 activity [154,155]. The crude extract inhibits the pre-infection activity of HSV-1. However, later research by Pongmuangmul et al. (2016) stated that chloroform extract of leaves of *C. nutans* contained monogalactosyl diglyceride (MGDG) and digalactosyl diglyceride (DGDG) [155]. Both MGDG and DGDG performed antiviral activities against HSV-1 and HSV-2 replication at the post-infection stage. The mechanism of anti-HSV-1 and anti-HSV-2 of MGDG and DGDG is unknown, but the anti-herpes simplex virus of monoglyceride was reported by the destruction of the viral envelope. 13^2^-hydroxy-(13^2^-*R*)-phaeophytin b, 13^2^-hydroxy-(13^2^-*S*)-phaeophytin a, and 13^2^-hydroxy-(13^2^-*R*)-phaeophytin a of *C. nutans* were shown to have anti-herpes simplex activity. Their inhibitory activity affected the viral adsorption or penetration onto the host cells [130]. Phaeophorbide A from leaves parts of *C. nutans* inhibited the production of dengue virus RNA as well as the protein of the infected cells [130].

Subsequently, Echinacea was found to have benefits against antivirus, such as cold and flu. Echinacea is widely used as a preventive compound for infectious diseases in respiratory systems. Essentially, it is being utilized to decrease the side effects and time span of colds, flu, and upper respiratory tract diseases and help to stimulate the activity of the immune system. *E. purpurea* roots were known to have antiviral properties against human coronavirus HCoV-229E and influenza virus [32,37], while the upper part of the plants helps to prevent cells with rhinovirus type 39 (RV-39) [36]. In addition, *E. pallida var. angustifolia* root showed anti-rhinovirus (RV) and anti-influenza activity, and *E. angustifolia* root extract had moderate antiviral activities against influenza virus and rhinovirus. Some echinacea extracts were found to inhibit hemagglutinin and neuraminidase of influenza viruses [31,37]. Echinacea was found to show direct virucidal and bactericidal activity against pathogenic respiratory bacteria and viruses. It promotes the proinflammatory response of epithelial cells and tissues to various respiratory viruses and bacteria while modulating certain immune cell functions as well as reducing the excessive mucin secretion induced by rhinovirus [31].

Four plants, *Glycyrhhiza* urelensis, *Phyllanthus emblica*, *Camellia sinensis,* and *Cistus incanus,* showed antiviral activity against viruses that cause influenza and dermatology-related diseases. Roots of *Glycyrrhiza* plants are prescribed as licorices that contain medicinal compounds, glycyrrhizin. The glycyrrhizin has anti-influenza properties by inhibiting influenza virus replication, inhibiting influenza virus polymerase activity, and anti-varicella-zoster virus (VZV) [75,124]. There are two vital antiviral components of *G. uralensis*, which are glycyrrhizin, which inhibits replication of SARS-associated virus, and glycyrrhizic acid, which possesses antiviral activity against enterovirus (EV71) as well as coxsackievirus (CVA16) infections that cause hand, foot, and mouth disease [73,107]. *Phyllanthus* plant is a flowering plant that can be found in tropical and subtropical regions. Branches and leaves of *P. emblica* showed potent inhibitory activity against influenza strain A/WSN/33(H1N1) [65], while the roots showed potential antiviral properties against influenza virus H3N2 and enterovirus EV71 [66]. Sarisetyaningtyas et al. (2006) stated that *P. niruri* extract syrup could help control the clinical manifestation of varicella-zoster virus infection. *C. sinensis* (green tea) is a shrub-type plant with fragrant, yellow-centered, and white flowers [125]. *C. sinensis* extract contains catechins that possess antiviral properties against influenza A virus (H1N1), influenza A (H3N2), influenza B virus, and human papillomavirus (HPV) [67,77,156]. *Cistus incanus* (family Cistaceae) is an evergreen dwarf shrub herb with hairy-sticky branches and leaves [69]. CYSTUS052 and Cipp (polyphenol-enriched fraction) are two promising antiviral compounds found in *C. incanus.* CYSTUS052 protective effect was shown by the binding of polymeric polyphenol components to the influenza virus surface and inhibiting the binding of hemagglutinin to the cellular receptors [60]. According to Rebensburg et al. (2016), CYSTUS052 prevent the primary attachment of HIV-1 and HIV-2 onto the cell surface and also prevents their envelope protein from binding to heparin [70]. The mechanism of action is mainly on blocking the viral attachment to cells and selectively targeting the viral envelope glycoprotein [69].

**Figure 8:**
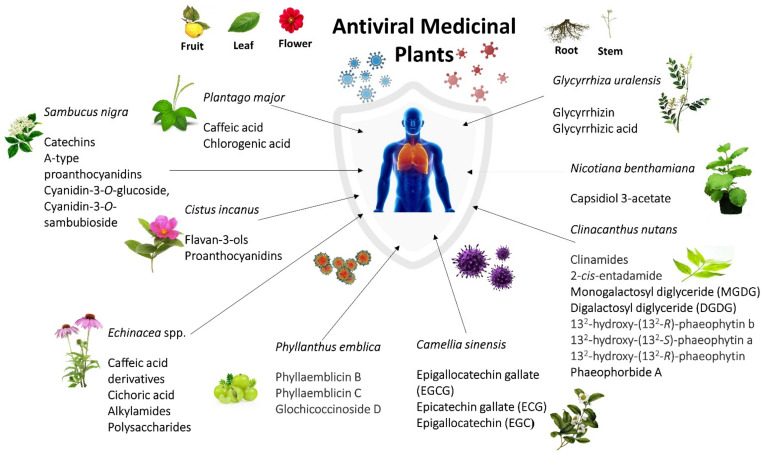
Overview of antiviral medicinal plants and their active compounds. The active compounds that were isolated were derived either from fruits, leaves, flowers, roots, or stems of plants.

*Plantago major* showed antiviral effects against viruses causing the common cold and sexually transmitted diseases. The phenolic compound, such as caffeic acid and chlorogenic acid of *P. major* had antiviral properties against a series of adenoviruses (ADV-3, ADV-8, ADV-11) and herpes simplex virus type 1 and 2. *P. asiatica* showed an antiviral effect against the respiratory syncytial virus [34,43]. Finally, among the most mentioned medicinal plants used as antivirus in this review is *N. benthamiana.* This plant expressed an anti-CHIKV monoclonal antibody (mAb) used in treating CHIKV infection in vivo and in vitro [137]. A plant-based protein known as ZIKV Envelope (PzE) was purified from leaves of *N. benthamiana* that provided treatment on mice against Zika viruses. It was revealed that PzE had the potential of inducing protective immunity of mice in vitro towards Zika virus infection. Table 7 and Figure 8 summarize the plants, their active compounds, and mechanisms of action on antiviral activity from the most promising medicinal plants.

## 8. Conclusions

The antiviral properties of 45 different medicinal plants worldwide were discussed. Most of the reviewed medicinal plants exhibited antiviral effects against influenza infection and dermatology-related viral infections, such as enterovirus, coxsackie virus, varicella-zoster virus, monkeypox virus, variola virus, and herpes simplex virus. We identified the 11 most promising plants with the most effects against different types of virus infections. We also highlighted the bioactive compounds that claimed to be responsible for the respective plants, as well as the mechanisms of action against the virus infections.

*S. nigra* and *C. nutans* were shown to possess the most potent antiviral effects. *Echinacea* spp (*E. purpurea, E. pallida, and E. augustofolia*) were found to be effective in treating colds and influenza. *G. urelensis*, *P. emblica*, *C. sinensis,* and *C. incanus* showed antiviral activity against viruses causing influenza and dermatology-related diseases. *P. major* revealed to own antiviral effects against viruses that cause the common cold and sexually transmitted diseases. Finally, *N. benthamiana* exhibits antiviral effects mostly against mosquito-borne viral diseases, such as chikungunya and Zika virus infection. These antiviral plants might provide a lead for the discovery of safe antiviral drugs to tackle various infections associated with different types of viruses. However, more preclinical studies of these medicinal plants are required to justify their efficacy against respective viruses. In general, this compilation will benefit the researchers and clinicians in narrowing down some targeted plant extracts and bioactive compounds for effective potential treatment against specific viral infections. More research is required to examine the efficacy of antiviral medications derived from traditional medicinal plant therapies, as most of these treatments have shown promising antiviral mechanisms of action.

## Figures and Tables

**Table 1:** Mechanism of action of common antiviral drugs.

Antiviral Drugs	Mechanism of Actions	Viruses
Acyclovir, Valacyclovir	Valacyclovir and valganciclovir are hydrolyzed with the presence of esterases secreted from the intestine and liver into nucleoside analogs, acyclovir, and ganciclovir, respectively [16,17]. Acyclovir and ganciclovir go through phosphorylation, catalyzed by intracellular and viral kinase into acyclovir-triphosphate and ganciclovir, respectively. Both triphosphate molecules act as substrates for viral DNA polymerization, compete with deoxyguanosine triphosphate (dGTP) and inhibit the elongation of the viral DNA chain [15,16].	HSV-1, HSV-2, and VZV
Ganciclovir, Valganciclovir		Cytomegalovirus (CMV) and HSV-1, HSV-2
Amantadine, Rimantadine	Interrupts the ion-channel activity of the M2 protein of influenza A by deforming the subunits of the channel [18]. The interruption inhibits the uncoating of viral RNA, thus preventing viral replication [19].	Influenza A
Zidovudine (ZDV), Lamivudine (3TC)	Both antiretroviral drugs work synergically through phosphorylation into zidovudine and lamivudine triphosphate, respectively, in host cells. Both triphosphate anabolites inhibit the enzyme for HIV reverse transcription and polymerization of hepatitis B virus, thus preventing the viral DNA elongation and viral multiplication [20,21].	HIV and hepatitis B virus
Remdesivir	This nucleoside analog prodrug undergoes a tri-phosphorylation process into remdesivir triphosphate. The nucleoside triphosphate embodies the new strand as the substrate for SARS-CoV-2 RNA-dependent RNA polymerase [22].	SARS-CoV-2
Nirmatrelvir	Also known as PF-07321332, a modified version of PF-00835231, hydroxymethyl ketone derivative. Nirmatrelvir inhibits the 3CL protease activity, an active drug target of SARV-CoV-2 that stimulates the cleave of the viral protein polypeptide bond [23].	SARS-CoV-2

**Table 2:** Medicinal plants possessing antiviral activity causing common cold.

Plant Name	Parts Used	Plant Extract	IC_50_/ED_50_/EC_50_/CC_50_/MIC_100_	Assay	Cell Line Used	Mechanism of Action	Outcome
*Echinacea pallida var. angustifolia* [31]	Root	70% ethanol	MIC₁_00_ = 62 μg/mL)	Viral plaque	Vero, H-1 and BEAS-2B	Inhibition of rhinovirus replication	Plant showed anti-rhinovirus activity
		Ethyl acetate	MIC₁_00_ = 85 μg/mL				
		Hexane	MIC₁_00_ = 69 μg/mL				
*Echinacea purpurea* [32]	Aerial parts and root	*v*/*v* 65% ethanol	IC_50_ = 3.2 μg/mL	MTT	Huh-7, Vero, and Vero E6	Inhibition of HCoV-229E penetration into the cell and progeny shedding	HCoV-229E virucidal activity
*Sambucus Formosana* Nakai [33]	Stem	Ethanol	IC_50_ = 1.17 ± 0.75 μg/mL	Virus yield reduction	LLC-MK2	Inhibition of binding of HCoV-NL63 with Sai cell-surface receptor	HCoV-NL63 virucidal activity
			IC_50_ = 4.67 ± 1.21 μg/mL	Viral plaque			
			IC_50_ = 15.75 ± 6.65 μg/mL	Virus attachment			
			CC_50_ = 180.62 ± 63.04 μg/mL	MTT			
*Plantago asiatica* [34]	Whole plant	Aqueous	CC_50_ = 938.43 μg/mL EC_50_ = 39.82 μg/mL SI value = 23.5	Cell cytotoxicity, standard viral plaque, RSV-GFP virus replication inhibition	Human epithelial type 2: HEp2 with HeLa contaminant and A549	Reduced RSV replication, RSV-induced cell death, RSV gene transcription, RSV protein synthesis, blocked syncytia formation	Antiviral therapy for respiratory syncytial virus (RSV) infection
*Clerodendrum trichotomum* [34]	Whole plant	Aqueous	EC_50_ = 27.95 μg/mL CC_50_ = 764.17 μg/mL. SI value = 27.3	Cell cytotoxicity, standard viral plaque, RSV-GFP virus replication inhibition	Human epithelial type 2: HEp2with HeLa contaminant and A549	Reduced RSV replication, RSV-induced cell death, RSV gene transcription, RSV protein synthesis, blocked syncytia formation	Antiviral therapy for respiratory syncytial virus (RSV) infection

**Table 3:** Medicinal plants possessing antiviral activity against influenza.

Plant Name	Parts Used	Plant Extract	IC_50_/ED_50_/EC_50_/CC_50_	Assay	Cell Line	Mechanism of Action	Outcome
*Punica granatum*	Peel [54]	80% ethanol	IC_50_ = 6.45 μg/mL CC_50_ = 55.66 μg/mL SI value = 8.63	Cytotoxicity, Cytopathic effect reduction	MDCK cells	Inhibition of influenza virus replication	Plant has high inhibitory effect on Influenza A virus replication in vitro
	Fruit [55,56]	Polyphenol extract		Real-time PCR, viral plaque, TCID50, MTT		Changed viral surface glycoproteins and promoted damage to virion integrity	Plant exhibit virucidal effect on Influenza A virus
*Geranium sanguineum* [57,58]	AerialRoots	Polyphenol-rich methanol and ethanol extract	EC_50_ = 3.6 μg/mL IC_50_ = 64 μg/mL	Cytopathogenic effect reduction	MDCK cells	Inhibited the CPE of Influenza A/Rostock virus in CEF cells	Plant extract showed virucidal effect on Influenza A/Rostock virus
			IC_50_ = 72 μg/mL		CEF cells		
*Echinacea purpurea* [37]	Roots	Aqueous fraction	MIC_100_ = 2.2–2.5 μg/mL	Cytopathogenic effect reduction, viral plaque	Vero, H-1, and BEAS-2B cell line	Eliminated viral cytopathic effect	Plant root contains a potent water-soluble antiviral ingredient
*Echinacea pallida var. angustifolia* [37]	Roots	Ethanol 55% extract	MIC_100_ = 33.5 μg/mL	Cytopathogenic effect reduction, viral plaque	Vero, H-1, and BEAS-2B cell line	Eliminated viral cytopathic effect	Plant root contains a potent water-soluble antiviral ingredient
		Ethyl acetate extract	MIC₁_00_ = 348 μg/mL				
*Cistus incanus* [59,60]	Dinstinct variety of *Cistus incanus*	CYSTUS052 extract	Effective dose = 50 μg/mL	Aerosol formulation treatment, plaque reduction, hemagglutination	MDCK cells	Prevented adsorption of the Influenza A virus to host cells	Plant extract reduced 90% Influenza A viral plaque formation
					Inbred female Balb/c and C57Bl/6 mice (age of 6–8 weeks)	Protected the animals against clinical disease symptoms caused by Influenza A virus infection	CYSTUS052-treated mice did not develop disease after being infected with the Influenza A
*Glycyrrhiza uralensis, Glycyrrhiza inflate* and *Glycyrrhiza glabra* (Licorice) [61]	Root and rhizomes	Glycyrrhizin	IC_50_ = 0.0081 μg/mL	Cell viability, CPE reduction, Virus yield reduction	Natural killer (NK) cells, Human peripheral blood mononuclear cells (PBMC)	Reduced H5N1-induced cytokine expression, H5N1-induced caspase activation, and H5N1-induced apoptosis	Glycyrrhizin may develop into potential anti-influenza drug
*Chaenomeles sinensis* [62,63]	Fruit	Polyphenol-rich extract, CSD 3	IC_50_ = 0.3 μg/mL	Plaque, hemagglutination, hemolysis	MDCK cells	Reduced cell-viral binding, hemagglutination, and hemolytic activities and infectivity of Influenza A/Udorn/72(H3N2)	Reduced primary transcription of Influenza A/Udorn/72(H3N2) virus-infected MDCK cells.
*Sambucus nigra* [64]	Fruit	Fruit juice at pH 4.4 ± 0.1	IC_50_ = 6000 ± 800 μg/mL CC_50_ = 770 ± 60 μg/mL	Cytotoxicity, plaque reduction	MDCK cells	Suppressed viral entry, restrained viral transmission from cell to cell, and modulated the release of cytokines	Plant’s fruit exhibited multiple modes of therapeutic action against influenza infection
			CC_50_ = 810 ± 30 μg/mL		A549 cells		
*Phyllanthus emblica*	Branches and leaves [65]	1,2,3,4,6-penta-O-galloyl-β-D-glucose (PGG) dissolved in DMSO	CC_50_ = 29.59 ± 4.32 μg/mL EC_50_ = 2.36 ± 0.29 μg/mL SI value = 12.54	Water-soluble tetrazolium-1, Plaque-forming unit (PFU)	MDCK cells, A549 cells	Inhibited hemagglutination of chicken red blood cells induced by Influenza A virus, reduced virus budding and progeny virus release from infected cells	PGG as a promising antiviral agent against influenza A virus
	Roots [66]	Fraction containing phyllaemblicin B	CC_50_ = 6.9 ± 0.9 μg/mL IC_50_ = 2.6 ± 0.7 μg/mL	Cytopathic end-point, Cytotoxicity	MDCK cells, Vero cells	Exerted inhibitory effect on Influenza A H3N2 strain	Both plant fractions showed potential anti-viral activities against Influenza A H3N2 strain
		Fraction containing glochicoccinoside D	CC_50_ =13.4 ± 2.1 μg/mL IC_50_ = 4.5 ± 0.6 μg/mL				
*Camelia sinensis* [67]	Leaves	Cathecin extract of (−)-epigallocatechin gallate (EGCG)	EC_50_ = 22–28 μg/mL CC_50_ = 275.4 ± 22.8 μg/mL	Plaque inhibition, Virus growth inhibition, Hemagglutination inhibition, Neuraminidase inhibition, MTT	MDCK cells	Inhibited hemagglutination inhibition activity, suppressed viral RNA synthesis, inhibited neuraminidase activity, inhibited viral attachment, altered viral and cell membrane	Inhibitory potential on Influenza A virus was shown in order of ECGC > ECG > EGC
		Cathecin extract of (−)-epicatechin gallate (ECG)	EC_50_ = 22–40 μg/mL CC_50_ = 525.9 ± 30.7 μg/mL				
		Cathecin extract of (−)-epigallocatechin (EGC))	EC_50_ = 309–318 μg/mL CC_50_ = 1233.1 ± 44.9 μg/mL				

**Table 4:** Medicinal plants possessing antiviral activity causing sexually transmitted disease.

Plant Name	Parts Used	Plant Extract	IC_50_/ED_50_/EC_50_/CC_50_	Assay	Cell Line	Mechanism of Action	Outcome
*Clinacanthus nutans* [87]	Leaves	n-hexane	IC_50_ = 32.05 µg/mL, 72.62 μg/mL CC_50_ = 1600 μg/mL	Plaque reduction, MTT	Vero	Inhibited HSV-1 and HSV-2 viral plaque formation	n-hexane and dichloromethane extracts exhibited better antiviral activity against HSV-1 than methanol extract
		Dichloro-methane	IC_50_ = 44.50 ± 2.66 µg/mL, 65.19 µg/mL CC_50_ = 869 μg/mL				
		Methanol	IC_50_ = 64.93 µg/mL, 65.13 μg/mL CC_50_ = 1600 μg/mL				
*Clinacanthus siamensis* [87]	Leaves	n-hexane	IC_50_ = 60.00 µg/mL, 46.52 µg/mL CC_50_ = 1600 μg/mL	Plaque reduction, MTT	Vero	Inhibited HSV-1 and HSV-2 viral plaque formation	Methanolic extract possessed the greatest anti-HSV-1. n-hexane and dichloromethane extracts exhibited the best anti-HSV-2 in vitro
		Dichloromethane	IC_50_ = 55.69 ± 4.41 µg/mL, 49.63 µg/mL CC_50_ = 194 μg/mL				
		Methanol	IC_50_ = 37.39 µg/mL, 72.64 µg/mL CC_50_ = 1600 μg/mL				
*Cistus incanus* [59,70]	Dinstinct variety of *Cistus incanus* (CYSTUS052 extract)	Aqueous (boiled)	EC_50_ = 8.06 μg/mL CC_50_ = 250 μg/mL	Time-of-addition (TOA), Virus attachment, Virus capture, MTT	HEK293T, H9, and LC5	Blocked primary virus attachment to cells by selective targeting of the viral envelope glycoproteins	Exhibited broad antiviral activity with low risk of virus resistance
		Polyphenol- enriched fraction	CC_50_ = 1200 μg/mL				Plant fraction possessed antiviral activity on HIV-1_LAI_
*Plantago major* [43]	Whole plant	Pure compound (caffeic acid)	EC_50_ = 15.3 µg/mL, 87.3 µg/mL SI value = 671, 118 CC_50_ = 10,293 µg/mL	XTT	BCC-1/KMC	Inhibited HSV virus replication	Caffeic acid possessed the best anti-HSV viral activity than chlorogenic acid
		Pure compound (chlorogenic acid)	EC_50_ = 47.6 µg/mL, 86.5 µg/mL SI value = 83.9, 46.2 CC_50_ = 3995 µg/mL				
*Polygonum minus* [88]	Leaves, stem	Methanol	LC_50_ leaves _=_ 875 µg/mL LC_50_ stem = 95 µg/mL	Virus attachment	Vero	Inhibited HSV-1 attachment	0.1 LC_50_ gave higher cell survival

**Table 5:** Medicinal plants possessing antiviral activity causing by dermatology-related disease.

Plant Name	Parts Used	Plant Extract	IC_50_/ED_50_/EC_50_/CC_50_	Assay	Cell Line	Mechanism of Action	Outcome
*Sarracenia purpurea* [96]	Whole plant	Juice	CC_50_ = 70–75 µg/mL SI value = 5–7	VACV plaque	RK-13	Prevented replication of monkeypox virus and variola virus	*Sarracenia purpurea* acted as effective inhibitor of poxvirus replication
*Clinacanthus nutans* [97,98]	Leaves	5% *C. nutans* cream	IC_50_ = 76 µg/mL	Double-blinded clinical trial	125 patients infected with VCV	Lowered the chance of developing clinical disease	Exhibited a positive curing effect against VZV infection
*Matricaria recutita* [99]	Whole plant	Essential oil	Not identified SI value = 20	Plaque reduction	RC-37	Interacted with the viral envelope and glycoproteins	Reduced the infectivity of the HSV-2 virus
*Aloe vera* [100,101]	Leaves	Hot glycerin extract	CC_50_ = 3238 µg/mL IC_50_ = 428 µg/mL SI value = 7.56	Cytotoxicity, plaque reduction	Vero	Inhibited HSV replication pre-attachment of virus on the cell	Showed significant inhibitory effect on HSV
			IC_50_ = 536 µg/mL SI value = 6.04			Inhibited HSV replication post attachment of virus replication	
*Cornus canadensis* [102]	Leaves	Ethanol	EC_50_ = 9 μg/mL	Plaque reduction, cytotoxicity	Vero	Inhibited the lysis plaque, inhibited virus absorption	Exhibited as the potent virus absorption inhibitors
*Lysimachia mauritania* [103]	Whole plant	Ethanol	IC_50_ = 26.09 µg/mL	Plaque reduction, cell viability	MRC-5	Inhibited the replication of varicella-zoster virus	Showed potent inhibitory effects on VZV gene expression and replication
*Mentha haplocalyx* [104]	Whole plant	Aqueous	IC_50_ = 70.3 μg/mL	Cytotoxicity, MTT, immunoblotting	Vero	Blocked viral infection proinflammatory response	Showed antiviral and anti-inflammatory activities
*Camellia sinensis* [105]	Leaves	Polyphenon E (poly E)	Concentration 1 and 5 µg/mL Concentration 10, 25, and 50 µg/mL	Immunofluorescence	TCL-1	Inhibited growth of HPV-immortalized cervical epithelial (TCL-1) cells	All compounds showed inhibitory response on growth of HPV and poly E
		Epigallocatechin gallate (EGCG)					
*Hibiscus sabdariffa* [106]	Leaves (red and green leaves)	Ethanol	Concentration of 10 and 15 mg/mL	Cytotoxicity	Hep-2	Inhibited measles virus replication	Showed antiviral activities on pre and post-inoculative treatment
*Glycyrrhiza uralensis* [107,108]	Root and stolon	Aqueous	IC_50_ = 0.056 μg/mL	XTT	Human foreskin	Suppressed EV71 replication	Showed antiviral activity against EV71 and CVA16 infection
		Glycyrrhizic acid	200 μg/mL	MTT, plaque forming	Vero	Blocked viral replication of EV71 and CVA16	Inhibited EV71 and CVA16 replication
*Phyllanthus emblica* [66]	Roots	Fraction containing glochicoccinoside D	IC_50_ = 2.6 ± 0.7 μg/mL	Cytopathic end-point, Cytotoxicity	MDCK, Vero	Inhibited EV71	Showed potential anti-viral activities against EV71
		Fraction containing phyllaemblicin C	IC_50_ = 2.6 ± 0.8 μg/mL				

**Table 6:** Medicinal plants possessing antiviral activity causing mosquito-borne viral disease.

Plant Name	Part Used	Plant Extract	IC_50_/ED_50_/EC_50_/CC_50_	Assay	Cell Line Used	Mechanism of Action	Outcome
*Alternanthera philoxeroids* [127]	Leaves	Coumarin based	TD_50_ = 535.91	MTT	C6/36	Inhibited C6/36 cell lines and dengue virus	Petroleum ether extract had the strongest inhibitory effect on dengue virus
		Petroleum ether	ED_50_ = 47.43				
*Cladosiphon okamuranus* [128]	Whole plant	Fucoidan	Concentration of 10 μg/mL	Focus-forming	BHK-21	Inhibited virus infection	Fucoidan reduced DENV-2 infectivity by 20% at 10 μg/mL
*Clinacanthus nutans*	Aerial part [129]	80% ethanol	IC_50_ 31.04 μg/mL	Anti-inflammatory, anti-dengue, immune-modulating activity	Naïve Huh-7	Anti-inflammatory, anti-dengue virus and immune-modulating activity	Possessed moderate anti-dengue virus activity
	Leaves [130,131]	Chloroform	CC_50_ of 25 μg/mL	MTT, immunofluorescence	C6/36, A549	Inhibited dengue viral 2 in pre-entry replication step and suppressed PGE2 production	Showed virucidal activity against dengue virus 2
*Sambucus nigra* [132]	Leaves and flowers	Methanolic	400 µg/mL	Viral plaque, indirect immunofluorescence	BHK-21 and Vero	Protected cell monolayers pre-treated cells before dengue virus-2 infection	Exhibited anti-DENV-2 activity on pre-incubated cells before dengue virus-2 infection
*Carica papaya*	Leaves [133,134]	Chloroform	CC_50_ = 1000 μg/mL EC_50_ = 1000 μg/mL SI value = 1	Plaque forming, cytotoxicity, anti-DENV2	LLC-MK2	Inhibited DENV2 growth	Possessed promising anti-dengue properties
		Aqueous	CC_50_ = 10437 μg/mL IC_50_ = 137.6 µg/mL SI value = 75.85	MTT, foci forming unit reduction (FFURA)	Vero	Inhibited the virus replication, decreased number of dengue viral foci	
*Picrorhiza kurroa*	Whole plant [135]	Aqueous	Min concentration to inhibit the plaque = 10 μg/mL	Viral plaque, helicase, protease	The monkey kidney cells, Vero	Inhibited viral attachment, inhibited helicase and protease activities	Showed virucidal activity on Chikungunya virus
*Ocimum tenuiflorum*							
*Terminalia chebula*							
*Rhapis excelsa* [136]	Leaves	Chloroform	EC_50_ = 29.9 ± 0.9 μg/mL CC_50_ = 161.5 ± 19.2 μg/mL	Cytopathic effect inhibition and cytotoxicity	African monkey kidney epithelial (Vero)	Showed cytopathic effect, inhibitory activity on Vero cells and reduction in the Chikungunya viral load	Showed virucidal activity on Chikungunya virus
*Vernonia amygdalina* [136]	Leaves	Ethyl acetate	EC_50_ = 32.4 ± 1.3 μg/mL CC_50_ = 165.5 ± 9.2 μg/mL		African monkey kidney epithelial (Vero)		
*Nicotiana benthamiana* (Wild type)	Leaves	CHKVmab extract	EC_50_ = 390.8 μg/mL	Plaque reduction	Vero (ATCC, CCL-81)	Neutralization activity against CHIKV	Plant monoclonal antibodies have the potential to be used as effective treatment to prevent CHIKV infection
*Nicotiana benthamiana* (glycoengineered) [137]			EC_50_ = 130.5 μg/mL				
*Aphloia theiformis* [138]	Aerial parts	Aqueous	CC_50_ = 3000 µg/mL IC_50_ = 100 µg/mL SI value = 30	Plaque-forming, Immunofluorescence	Vero and human-derived Huh7.5 hepatoma	Prevented the viral entry into host cells	Promising sources of naturally derived antiviral compounds to prevent ZIKV
*Psiloxylon mauritianum* [139]	Fresh aerial parts	Aqueous	CC_50_ = 1044 ± 106.2 μg/mL	MTT, genotoxicity, viral inactivation, Time-of-drug-addiction	Vero	Inhibited early steps of the viral replication	Showed antiviral activity against historical and contemporary strains of ZIKV
			CC_50_ = 657 ± 15.7 μg/mL		A549		
			CC_50_ = 353 ± 84.4 μg/mL		Human primary keratinocytes		
			CC_50_ = 820 ± 26.5 μg/mL		Fibroblast (FMa)		
			IC_50_ = 19.5 ± 4.8 μg/mL SI value = 53.5				

**Table 7:** Medicinal plants, their active compounds, and mechanism of action on antiviral activity.

Plant	Active Compound (s)	Mechanism of Action
*Sambucus nigra*	Catechins and A-type pro-anthocyanidins, cyanidin-3-*O*-glucoside, cyanidin-3-*O*-sambubioside	Bind to surface of influenza virus and prevent the influenza virus from entering and replicating in the host cell [152].
		Plant lectins bind to host cell membranes and prevent the influenza virus hemagglutinin’s attachment to host cells [39].
		Block the ability of HIV virions to infect host cells [157].
*Clinacanthus nutans*	Clinamides and 2-*cis*-entadamide	Promote down-regulation of IFN-γ and exhibit immune-modulating activities [129].
	monogalactosyl diglyceride (MGDG) and digalactosyl diglyceride (DGDG).	Anti-HSV activities at post-infection stage [155].
	13^2^-hydroxy-(13^2^-*R*)-phaeophytin b, 13^2^-hydroxy-(13^2^-*S*)-phaeophytin a, and 13^2^-hydroxy-(13^2^-*R*)-phaeophytin	Affected the viral adsorption and penetration of HSV into host cells [130].
	Phaeophorbide A	Inhibit the making of dengue virus RNA and protein in infected cells [130].
*Echinacea* spp.	Caffeic acid derivatives and cichoric acid	Enhanced innate immunity through activation of the neutrophils, macrophages, polymorphonuclear leukocytes (PMN), and natural killer (NK) cells [37,158].
	Caffeic acid derivatives, alkylamides, polysaccharides	Suppress cytokine and chemokine production from human blood monocytes stimulated by influenza viruses [159,160].
*Plantago major*	Caffeic acid, chlorogenic acid	Inhibit replication of HSV-1, HSV-2, ADV-3 and ADV-11 [43].
*Glycyrrhiza uralensis*	Glycyrrhizin	Inhibition of influenza virus replication by inhibiting virus polymerase activity [75].
	Glycyrrhizic acid	Targeting early infection of coxsackievirus A16 on Vero cells to deactivate or inhibit coxsackievirus A16 infection [107].
*Phyllanthus emblica*	Phyllaemblicin B, phyllaemblicin C, and glochicoccinoside D	Displayed anti-viral activities and inhibitory activities against influenza A virus (H3N2), Enterovirus (EV71), coxsackievirus B3 and HSV-1[66].
*Camellia sinensis*	Epigallocatechin gallate (EGCG)	Inhibits hemifusion events between virus particles and the cellular membrane by reducing the viral membrane integrity, thereby resulting in the loss of the cell penetration capacity of the influenza virus [161].
	Epicatechin gallate (ECG) and EGCG	Inhibits neuraminidase activity and blocks the function of viral neuraminidases of the influenza virus [162].
		Exhibits hemagglutination inhibition activity [67].
		Suppresses viral RNA synthesis in MDCK cells [67].
	Epigallocatechin (EGC)	Inhibits neuraminidase activity and blocks the function of viral neuraminidases of the influenza virus [67].
*Cistus incanus*	flavan-3-ols and proanthocyanidins	Bind to the virus surface and inhibit the binding process of hemagglutinin to cellular receptors [60]
		Prevent primary attachment of the HIV-1 and HIV-2 onto the cell surface [70].
		Blocking the viral attachment to cells and selective targeting the viral envelope glycoprotein [69].
*Nicotiana benthamiana*	capsidiol 3-acetate	Inducing self-defense mechanism in *Nicotiana benthamiana* against Potato virus X infection [163].

## Data Availability

Not applicable.

## Abbreviations

IC_50_	50% inhibitory concentration
EC_50_	Half maximal effective concentration
CC_50_	50% Cytotoxic concentration
ED_50_	Median effective dose
TD_50_	Median toxic dose
MIC_100_	Minimum inhibitory concentration at 100 µm
MOI	Multiplicity of infection
